# Intraspecific evolutionary relationships among peregrine falcons in western North American high latitudes

**DOI:** 10.1371/journal.pone.0188185

**Published:** 2017-11-17

**Authors:** Sandra L. Talbot, George K. Sage, Sarah A. Sonsthagen, Meg C. Gravley, Ted Swem, Jeffrey C. Williams, Jonathan L. Longmire, Skip Ambrose, Melanie J. Flamme, Stephen B. Lewis, Laura Phillips, Clifford Anderson, Clayton M. White

**Affiliations:** 1 U.S. Geological Survey Alaska Science Center, Anchorage, Alaska, United States of America; 2 Fairbanks Fish and Wildlife Field Office, U. S. Fish and Wildlife Service, Fairbanks, Alaska, United States of America; 3 Alaska Maritime National Wildlife Refuge, U. S. Fish and Wildlife Service, Homer, Alaska, United States of America; 4 Los Alamos National Laboratory, Los Alamos, New Mexico, United States of America; 5 Yukon-Charley River National Preserve and Gates of the Arctic National Park and Preserve, National Park Service, Fairbanks, Alaska, United States of America; 6 Migratory Bird Management, U. S. Fish and Wildlife Service, Juneau, Alaska, United States of America; 7 Alaska Regional Office, National Park Service, Anchorage, Alaska, United States of America; 8 Falcon Research Group, Bow, Washington, United States of America; 9 Department of Plant and Wildlife Sciences and Monte L. Bean Life Science Museum, Brigham Young University, Provo, Utah, United States of America; University of Arkansas Fayetteville, UNITED STATES

## Abstract

Subspecies relationships within the peregrine falcon (*Falco peregrinus*) have been long debated because of the polytypic nature of melanin-based plumage characteristics used in subspecies designations and potential differentiation of local subpopulations due to philopatry. In North America, understanding the evolutionary relationships among subspecies may have been further complicated by the introduction of captive bred peregrines originating from non-native stock, as part of recovery efforts associated with mid 20^th^ century population declines resulting from organochloride pollution. Alaska hosts all three nominal subspecies of North American peregrine falcons–*F*. *p*. *tundrius*, *anatum*, and *pealei*–for which distributions in Alaska are broadly associated with nesting locales within Arctic, boreal, and south coastal maritime habitats, respectively. Unlike elsewhere, populations of peregrine falcon in Alaska were not augmented by captive-bred birds during the late 20^th^ century recovery efforts. Population genetic differentiation analyses of peregrine populations in Alaska, based on sequence data from the mitochondrial DNA control region and fragment data from microsatellite loci, failed to uncover genetic distinction between populations of peregrines occupying Arctic and boreal Alaskan locales. However, the maritime subspecies, *pealei*, was genetically differentiated from Arctic and boreal populations, and substructured into eastern and western populations. Levels of interpopulational gene flow between *anatum* and *tundrius* were generally higher than between *pealei* and either *anatum* or *tundrius*. Estimates based on both marker types revealed gene flow between augmented Canadian populations and unaugmented Alaskan populations. While we make no attempt at formal taxonomic revision, our data suggest that peregrine falcons occupying habitats in Alaska and the North Pacific coast of North America belong to two distinct regional groupings–a coastal grouping (*pealei*) and a boreal/Arctic grouping (currently *anatum* and *tundrius*)–each comprised of discrete populations that are variously intra-regionally connected.

## Introduction

Clarification of the relationship between phenotypic and molecular characters is particularly relevant to studies involving northern high-latitude avian species, as many vertebrate populations there represent the leading edge of post-Pleistocene expansions from temperate glacial refugia [1]. Such populations are often little differentiated, since insufficient time has passed for neutral genetic markers to coalesce [2], leading to discordance between morphological and genetic variation at neutral genetic markers. Molecular genetic phylogenies have failed to support many avian subspecies designations that are based on phenotypic characters [3,4], including designations in species of raptor that occupy high latitude habitats [5,6], although the criteria for designating subspecies based on genetic characteristics vary across investigators [7]. Nevertheless, difficulties in understanding underlying genetic and environmental bases of phenotypic characters have contributed to inconsistencies when making evolutionary inferences based on either character class, and this has also contributed to controversy involving subspecies designations that are based partially or largely on melanin-based plumage traits [5], which can vary with individual body condition, age, and exposure to oxidative stress induced by environmental factors [8].

Differences in levels of melanism, which can be due to variation at one or a number of genes, can also emerge from both neutral and adaptive evolutionary processes, such as genetic drift [9] for the former, and divergent and disruptive evolution, preferential selection for mates displaying a specific color [10], and morph-specific variation in immunity as well as pathogen pressure [11] for the latter. For example, a single non-synonymous change in the melanocortin-1 receptor gene (*MC1R*), which codes for primary plumage color in avian species, is perfectly associated with the presence of melanism in two Arctic species, the snow goose (*Chen caerulescens*), and Arctic skua (*Stercorarius parasiticus*), in which polymorphisms in color apparently influence mate choice [12]. Although white and melanistic snow geese were once thought to comprise different species or subspecies [13], the *MC1R* genotype-phenotype association in snow geese, as well as skuas, is specific to the *MC1R* locus and does not reflect shared demographic history of populations containing different morphs [12]. Similarly, a single nucleotide polymorphism (SNP) found in the *MC1R* gene is a determinant of leucistic captive [14] and wild [15] gyrfalcons (*Falco rusticolus*), a raptor species that occupies Arctic habitats, but for which variation in plumage color (from all-white to dark gray/brown) is not currently associated with subspecies designations [16–18]. In gyrfalcons, however, there is no evidence of assortative pairing based on plumage color, although there may be a relationship between plumage color and timing of egg-laying and reproductive output [15]. In contrast, even though subspecies designations in the red-tailed hawk (*Buteo jamaicensis*) in western North America are based in part on the presence of certain melanin-based plumage characteristics, there is no apparent correlation between specific variants of the *MC1R* gene and variation in plumage color [5].

Similar to red-tailed hawks, designations among the named subspecies of peregrine falcons (*F*. *peregrinus*) in North America are based in part on morphological characteristics, including plumage traits associated with melanism [19]. Prior population genetics studies suggest that these morphological characteristics do not fully predict the distribution of genetic variation among representative populations, at least within the Canadian portion of the species’ North American range [6,7]. It is unclear whether this discordance ensues wholly from historical and demographic processes associated with natural colonization, coupled with inadequate passage of time for coalescence [2], or from recovery efforts associated with the late 20^th^ century augmentation or reestablishment of extirpated peregrine falcon populations using non-native stock [20,21], or a combination of these and/or other factors.

**Fig 1:**
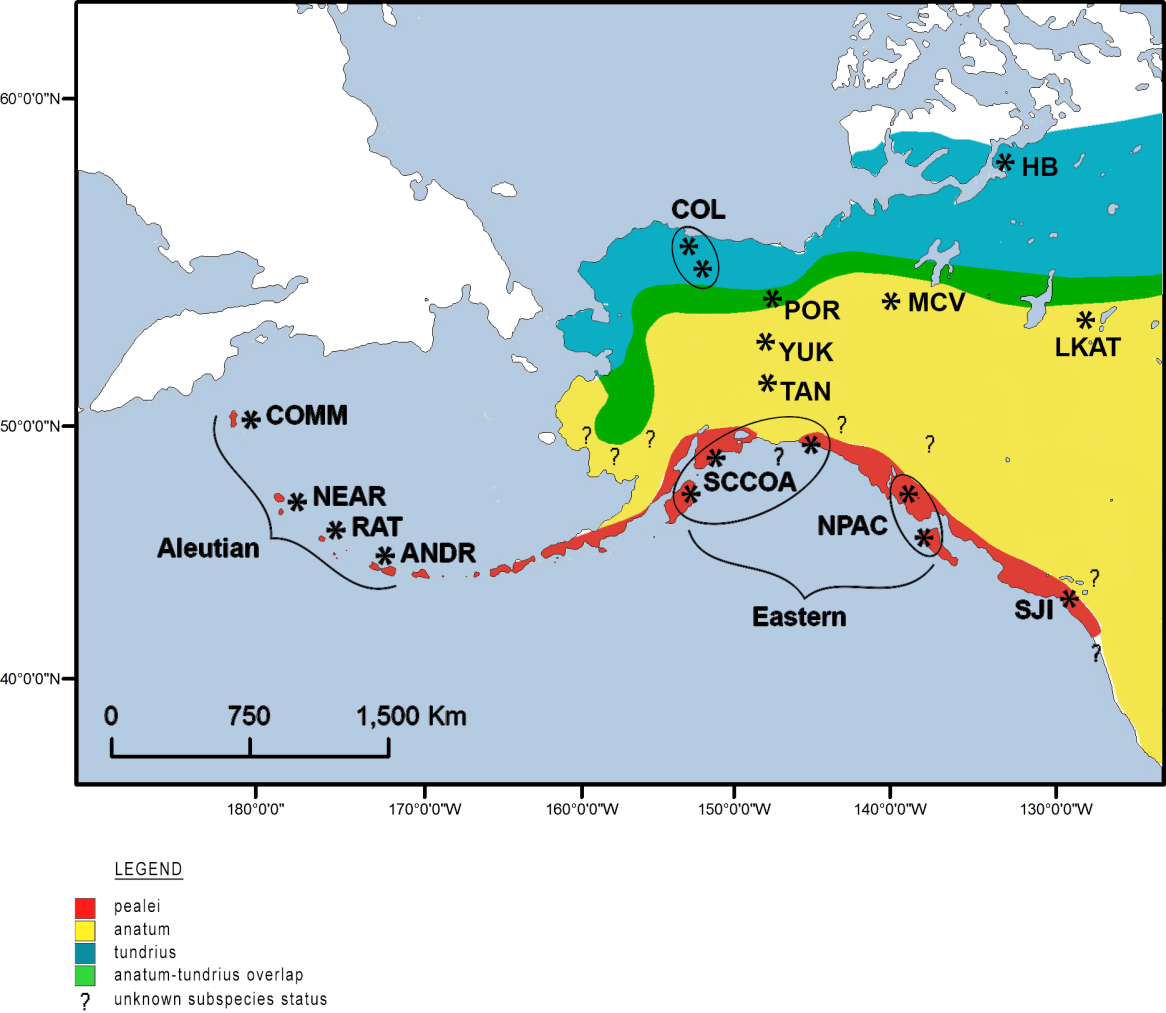
Map of peregrine falcon subspecies distributions in Alaska and Canada, and general sampling locales.

In North America, peregrines breed from Mexico to the middle Arctic and from the Pacific to the Atlantic coast. Morphological variation among peregrine falcons is sufficient that 19 subspecies have been described worldwide [22], three of which nest during summer months in high latitude North America. Peale’s falcon (*F*. *p*. *pealei* Ridgway 1873), the largest subspecies, is distributed in maritime habitats coastally from Washington State through the Aleutian Islands, Alaska, to the Commander Islands, Russia (Fig 1). Peregrines within the geographic distribution of *F*. *p*. *pealei* are characterized by dark, heavily pigmented plumage, a diet comprised mostly of alcids and procellarids, year-round residency, and relative stability of component populations when the other two North American subspecies declined due to reproductive failure resulting from organochloride contamination. Plumage coloration varies within the Peale’s falcon; peregrines occupying the Aleutian Islands are uniformly dark, whereas those from Haida Gwaii (formerly Queen Charlotte Islands) have grayed tones and show a greater range of variation [23].

The Nearctic subspecies (Fig 1), *F*. *p*. *tundrius* [23], is generally smaller and paler than the other two North American subspecies, and they exhibit highly migratory behavior. The breeding range of *F*. *p*. *tundrius* is in the Nearctic tundra region (herein, Arctic Alaska or Arctic Canada), from about 77° N south to treeline, east to Greenland and west to Cape Prince of Wales, Alaska, and individuals within the range of *F*. *p*. *tundrius* winter from Florida south to Argentina (38° S) and Chile (41° S). The third subspecies, *F*. *p*. *anatum* (Bonaparte 1838), has a continental distribution (Fig 1), and is considered intermediate between the other two subspecies in size, plumage color, and migratory behavior. Based on analysis of morphological characteristics of museum specimens, White [23] found that peregrine falcons in interior boreal habitats in Alaska are on average larger than peregrines occupying locales within the southwestern distribution of *F*. *p*. *anatum*, and darker than the pale, small, highly migratory peregrines occupying the Arctic (that is, peregrines within the distribution of *F*. *p*. *tundrius*). However, there is overlap in plumage characteristics and morphological measurements in Alaskan *F*. *p*. *tundrius* and *F*. *p*. *anatum* [19].

Studies conducted in the early part of the 20^th^ century suggest the peregrine falcon was never abundant in North America [20,24]. Reproductive failure due to organochloride pollution caused a severe decline in most temperate and high latitude North American peregrine falcon populations, starting in the late 1940s and continuing into the early 1970s [24]. This well-documented and unprecedented collapse extirpated peregrine falcons in many regions of North America [25], including major former strongholds such as the Appalachian region [26]. In some locales (e.g., the Yukon and Tanana rivers, Alaska [27]), localized human activity, such as road construction and take for falconry [26,27,28], may have exacerbated declines attributed to the pesticide 1,1,1-trichloro-2,2-bis (*p*-chlorophenyl) ethane (DDT). Insular, non-migratory populations, including *F*. *p*. *pealei* along the Pacific Coast of Alaska and British Columbia (with the exception of the population on Haida Gwaii) and along the Aleutian Island chain, were considered least affected [20,25]. By 1975, only 324 nesting pairs of peregrine falcons were confirmed on the entire North American continent [24] although numbers were probably larger by perhaps 25% (estimate based on personal experience of authors and others). Peregrines occupying boreal (*F*. *p*. *anatum*) and Arctic (*F*. *p*. *tundrius*) habitats were listed as endangered in 1970 under the Endangered Species Conservation Act of 1969 (P.L. 91–135, 83 Stat. 275), a law that preceded the Endangered Species Act of 1973. Among the three subspecies, *F*. *p*. *anatum* was the focus of the most intense recovery efforts.

Recovery of the peregrine falcon in North America proceeded after the ban of DDT for agricultural use in Canada in 1970, and the United States in 1972, and the subsequent, although gradual, decrease of the breakdown product 1,1-dichloro-2,2-bis (*p*-chlorophenyl) ethylene, DDE, in North American food chains. Recovery efforts in Canada and much of the contiguous United States that occurred between 1974 and 1999 included the introduction of captive-bred peregrine falcons to bolster remnant or replace extirpated populations [21,29]. Brown et al. [7] report that only native birds were released during the Canadian reintroduction efforts. However, this claim applies perhaps to only a portion of the Canadian reintroductions, begun in the early 1980s, that used only native-captured *F*. *p*. *anatum* to augment eastern Canadian populations [30]. Falcons released into portions of Canada and the continental United States prior to the mid-1970s were captive stock derived from seven different subspecies, including (in addition to the three North American subspecies) four subspecies found on other continents (Europe: *F*. *p*. *brookei*, *F*. *p*. *peregrinus*; South America: *F*. *p*. *cassini*; and Australia: *F*. *p*. *macropus* [21,30,31]). Further, peregrines released in the contiguous United States were known to have dispersed into Canada, where they bred [31]. Although monitored *F*. *p*. *tundrius* populations had declined by approximately 80%, this subspecies was downlisted from endangered to threatened in 1984 (49 *FR* 10520, March 20, 1984) and delisted in October 1994 (59 *FR* 50796, October 5, 1994). Populations of *F*. *p*. *anatum* were delisted in the United States on August 25, 1999 (64 *FR* 46541–46558).

In Alaska, which like Canada hosts all three nominal North American subspecies, recovery of peregrines occupying boreal and Arctic habitats commenced in the late 1970s, following the prohibition of DDT for agricultural purposes in 1972 [27,32]. Unlike elsewhere in North America, however, reintroductions were not conducted in Alaska; populations there were allowed to recover through natural recruitment and recolonization [19,20,33,34]. As a result, genetic assessment of relationships among Alaskan populations comprising the three named subspecies may be less confounded by the introduction of genetic signatures of peregrine falcons from other regions, as in Canada [7] and Scandinavia [35]. Nevertheless, it is not known whether peregrines released in the contiguous United States and Canada during the late 20^th^ century recovery effort dispersed into Alaska. As well, previous inferences associated with levels and distribution of genetic diversity within North American *F*. *p*. *pealei* [7] did not include information from falcons occupying the Aleutian Island chain, which represents the largest and most geographically isolated component of this subspecies’ distribution.

To provide a phylogeographic framework within which to assess the relationships among the named subspecies, we used genetic markers from the mitochondrial and nuclear genomes to investigate taxonomic relationships among the three North American subspecies occupying non-augmented northwestern-most locales. We focus on two central questions: 1) is the current subspecies taxonomy, which is based largely on plumage characteristics, predictive of the distribution of neutral gene variation within Alaskan peregrine falcons; and 2) what are the relationships among populations within named subspecies in western high latitude North America? We use sequence data from the control region of the mitochondrial DNA and fragment data from 11 polymorphic microsatellite loci to investigate relationships among maritime populations occupying the Aleutian Island chain, including the Commander Islands, Russia, and coastal North Pacific locales (*F*. *p*. *pealei*), as well as boreal (*F*. *p*. *anatum*) and Arctic habitats (*F*. *p*. *tundrius*) of Alaska. We also include comparative data from a population of falcons nesting on the San Juan Archipelago in western Washington State, a locale thought to comprise a contact zone between southeastern populations of *F*. *p*. *pealei*, and southern populations of *F*. *p*. *anatum* [6]. To infer levels of gene flow from potentially augmented populations, and, given the mid-to-late 20^th^ century decline in two of the three North American subspecies, to test for genetic signatures of recent demographic change against a background of more historical population change, we include data from falcons of western Canadian Arctic and boreal populations.

## Materials and methods

### Ethics statement

Samples included in this study were obtained from: A) specimens in museum collections (the Monte L. Bean Museum, Provo, Utah (n = 16) and the Museum of the Zoological Institute in St. Petersburg, Russia (n = 7); B) a single blood sample from the Bird Treatment and Learning Center, collected by veterinarians or other clinical staff with permission of State and Federal wildlife authorities; and C) blood, eggs, epithelial swabs, or feather samples collected as part of past and ongoing field research [27,32,36–40], all collected with appropriate Federal and State or Provincial permits. The latter include appropriate permits from the U. S. Fish and Wildlife Service (all collections in the United States) and the Canadian Wildlife Service (all collections in Canada), site-specific permits for collections on lands administered by the National Park Service-and the U. S. Fish and Wildlife Service’s National Wildlife Refuge-system, the Washington State Department of Fish and Game (the San Juan Island samples), and the Alaska Department of Fish and Game (for scientific collection in Alaska). All field sampling procedures (blood withdrawal) were reviewed prior to sample collection. Field activities subsequent to 1985, with the exception of those by U. S. Fish and Wildlife Service personnel, were conducted following appropriate Institutional Animal Care and Use Committee (IACUC), or equivalent, approval. U. S. Fish and Wildlife Service did not implement requirements for IACUC permitting until 2008; all field research conducted by U. S. Fish and Wildlife personnel (TS, SA, SBL) subsequent to 2008 were conducted under an appropriate IACUC. In all cases, blood withdrawal procedures followed recommendations by the Ornithological Council for handling wild birds and sampling tissues [41]. Year and location of sample collection are provided [42]. Samples are held at the U. S. Geological Survey Alaska Science Center but are not available for loan.

### Field techniques

We collected whole blood, buccal swabs, and/or feathers from adult nesting female and male peregrine falcons, or young in nests, from sites within the distribution of each subspecies (Fig 1). The Arctic subspecies, *F*. *p*. *tundrius*, is represented in Alaska by samples taken from the Sagavanirktok River (n = 1) and the Colville River (n = 46), pooled and designated as COL, and in western Canada by samples collected from Hope Bay, Northwest Territories (HB, n = 18, [38]). *F*. *p*. *anatum* in Alaska is represented by samples collected from sites along the Porcupine (POR, n = 9), Tanana (TAN, n = 9) and Yukon (YUK, n = 17) rivers, and in western Canada by samples collected from Alberta and Saskatchewan [38,40], mostly from the Lake Athabasca area (LKAT, n = 6), and the Mackenzie Valley (MCV, n = 8). The eastern portion within the distribution of *F*. *p*. *pealei* is represented by samples collected from 1) the North Pacific coast of North America, including the Alexander Archipelago in southeast Alaska, and Haida Gwaii Archipelago of British Columbia (NPAC, n = 11), and south central coastal Alaska, including locales near Yakutat, Kenai Fjords, and Kodiak Island (SCCOA, n = 13). The western distribution of *F*. *p*. *pealei* is represented by samples (n = 44) collected from across four island groups along the Aleutian island chain; these include the Rat (Amchitka and Buldir islands; RAT, n = 17), Andreanof (Kasatochi and Amatignak islands; ANDR, n = 17), Near (Attu Island; NEAR, n = 5) and Commander (COMM, n = 7) island groups. Peregrines sampled from the San Juan Islands (SJI, n = 15), thought to represent a contact zone between continental *F*. *p*. *anatum* and the North Pacific segment of *F*. *p*. *pealei* [6], are treated as a separate population not designated to a subspecies. Sample locales and acronyms are given in Fig 1, specific data associated with each sample are provided in [42].

Blood samples were obtained from either nestlings or falcons trapped using standard techniques (see [29]). Blood was taken from the brachial vein using non-heparinized needles and stored in 1mL of blood preservation buffer (0.1M Tris-HCl [pH 8.0], 0.1M EDTA, 0.1M NaCl, and 0.5% SDS; [43]). Buccal swabs were collected using techniques similar to Handel et al. [44], except after collection, swabs were stored in 500 μL of preservation buffer. Feathers and eggshell membranes were kept in coin envelopes stored in silica gel until DNA extraction. Samples were kept at room temperature until DNA was extracted, and thereafter stored at -80°C (for blood and swabs) or in silica gel in dry cabinets (feathers and eggshell membranes).

### Laboratory techniques

See S1 Methods for details of the laboratory analyses. Following extractions and quantification of DNA, fragment data were collected at 11 polymorphic loci ([7,45–47]) and nucleotide sequence data from a 559 base pair segment of domain 1 of the mtDNA control region, from peregrine falcons collected from 14 locales across Arctic, boreal, coastal Alaskan forests and the Aleutian Islands, the San Juan Islands in Washington State, and western Canada, following procedures outlined elsewhere [6,47].

### Data analyses

#### Phylogenetic analyses

An unrooted phylogenetic network was constructed for mtDNA control region haplotypes using NETWORK 4.610 (Fluxus Technology, Clare, United Kingdom) and the reduced median method [48].

#### Genetic variability and tests of neutrality

Details of analyses assessing levels of genetic diversity and neutrality at microsatellite loci and the mtDNA control region are provided in S1 Methods. Briefly, we quantified genetic variation and tested for neutrality in microsatellite loci and mtDNA control region sequences using a variety of computer programs routinely used to analyze genetic data [49–51]. We note that tests of selective neutrality of mtDNA control region sequence data (Fu's *F*s [52] and Tajima's *D* [53]) can also provide evidence of population expansion (see S1 Methods).

#### Population structure and regional differentiation

Details of analyses to assess levels of population and regional genetic structure are provided in S1 Methods. Significance of spatial variation in microsatellite allele frequencies between populations was assessed using F-statistics (F_*ST*_ and R_*ST*_) and their analogs ([54,55], respectively), using several computer programs routinely used to analyze genetic data [49–51]. Significance of F_*ST*_ and R_*ST*_ values were based on random permutation tests (n = 1,000); Critical α-values were adjusted using Bonferroni corrections. Because populations are represented by unequal sample sizes [56], we also estimated population differentiation based on the distribution of microsatellite alleles across populations, using Fisher’s combined probability test [57,58]; significance was judged based on random permutations tests (n = 1,000) across loci between populations and adjusting critical α-values using Bonferroni tests. We used the likelihood ratio test criterion in MODELTEST 3.06 [59] to determine the evolutionary distance model that best fit the mtDNA control region sequence data and applied those weighted distances to calculate population pairwise F_*ST*_ (ϕ_ST_; [60]) and test for significance [49]. Population differentiation estimated from the distributions of mtDNA haplotypes were based on the log-likelihood (G) test [56] with significance based on random permutation tests (n = 1,000). Microsatellite data were also analyzed using Bayesian cluster analyses [61,62] to detect the occurrence of population structure without *a priori* knowledge of putative populations.

We used hierarchical analyses of molecular variance (AMOVA, [60]) to test for significant geographic partitioning of *a priori* hypothesized regional units (subspecies), using ARLEQUIN 3.1 [49]. Calculations for mtDNA sequence data incorporated a distance matrix based on the model of evolution determined by MODELTEST that best fit the data, and were also conducted without applying a distance matrix. In addition to the *a priori* groups of testable subspecies hypotheses applicable to peregrine falcons of North America, we experimented with various *a posteriori* groups in AMOVA analyses for both marker types that tested relationships of certain populations to named subspecies (e.g., the placement of SCCOA and SJI relative to *anatum* and *pealei*). We assumed that the best geographic subdivisions were significantly different from random distributions and had maximum among group variance (Φ_CT_ values). We also examined regional interrelationships by constructing and visualizing a network based on Cavalli-Sforza and Edwards [63] genetic distances (C_SE_) among microsatellite loci and neighbor-joining methods [63–65]. Two analyses were conducted: 1) populations were analyzed separately, and 2) populations were pooled into assigned subspecies, except for *F*. *p*. *pealei*, which was partitioned into the Aleutian group and the eastern group, based on results of population differentiation analyses.

#### Estimation of levels and polarity in gene flow

We estimated the magnitude and polarity of evolutionary dispersal (gene flow) among populations and subspecies, using the maximum likelihood approach implemented in MIGRATE 3.0.3 [66,67]; details of the gene flow analyses are provided in S1 Methods. Migrate uses a coalescent approach to estimate gene flow rates (Nm) among populations, assuming a constant per-locus mutation rate (μ). Values calculated (see S1 Methods) included number of migrants (m) among populations per generation (4N_e_m) and number of female migrants per generation (N_f_m) for nuclear microsatellites and mtDNA sequences, respectively. Significance of asymmetry in gene flow was based on non-overlapping 95% confidence intervals generated from full models, in which composite measures θ (4N_e_μ or N_f_μ) and M (m/μ) were estimated individually from the data and allowed to vary among populations. For this analysis, HB and MCV, which showed no significant interpopulational structuring even though they have traditionally been ascribed to different subspecies, were pooled to represent Arctic Canada; similarly, POR, TAN and YUK were pooled to represent interior boreal Alaska and the different Aleutian Archipelago islands were pooled to represent the Aleutian Islands as a single group. Arctic Alaska is represented by COL.

#### Genetic signals of changes in population demography

Genetic evidence for recent demographic fluctuations was evaluated for the microsatellite loci using BOTTLENECK 1.2.02 [68], applying three models of mutation (the infinite allele model, IAM; [69]; the stepwise mutation model, SMM [70], and two-phase model, TPM [71]); parameters for the TPM followed published guidelines [72,73]. Significance was assessed using a Wilcoxon sign-rank test. Significant heterozygosity deficit values relative to the number of alleles indicate recent population growth, whereas heterozygosity excess relative to the number of alleles indicates a recent population bottleneck [68]. To assess recent population demography using sufficient numbers of representative individuals, we pooled data from TAN, POR, and YUK into a single population, and similarly pooled NEAR and COMM.

Evidence of more historical population change trends was evaluated using mtDNA sequence data, employing FLUCTUATE [74], which estimates a population growth parameter, *g*, incorporating coalescence theory. Positive values of *g* suggest population growth over deeper time frames; negative values suggest population decline. Because this method may be upwardly biased [74], we used *g* to indicate population growth if *g* > 3 SD(*g*). Fluctuations in historical population size were also inferred from Tajima’s *D* and Fu’s *F*_S_, generated using ARLEQUIN to test for locus neutrality. Additional details of these analyses are provided in S1 Methods.

## Results

### Genetic diversity, population, and regional differentiation

#### Microsatellite data

Standard genetic diversity metrics for each sampling locale for microsatellite data are summarized in Table 1. All loci were in HWE except two, NVHfp54 (χ2 = 46.24, df = 22, *P* = 0.002) and NVHfp92-1 (χ2 = 96.98, df = 28, *P* < 0.001), which differed significantly from HWE expectations due to deviations in 2 and 6 of the 14 populations, respectively. Analyses using MICRO-CHECKER 2.2.3 [75] suggested the homozygote excess observed in NVHfp92-1 (in TAN, MVC, COL, SJI, and COMM) may be attributable to null alleles, although homozygote excess was not observed in the remaining 11 populations. No population deviated from HWE overall, and we detected no evidence of significant linkage disequilibrium across locus pairs for any single population overall or within any population except SCCOA (3 instances, all involving NVHfp31; *P* = 0.014–0.048). Inbreeding coefficients for all populations were not significantly different from zero (Table 1). Average expected heterozygosity was highest in SCCOA and lowest in TAN, ANDR and POR; allelic richness [76] was highest in NPAC and lowest in ANDR (Table 1). When populations were pooled into subspecies, average expected heterozygosity and allelic richness was highest in *F*. *p*. *pealei* and lowest in *F*. *p*. *tundrius*. Microsatellite genotype data are accessioned at the USGS-ASC data repository [42].

**Table 1:** Genetic diversity, neutrality and growth indices for 11 microsatellite loci and mitochondrial control region sequence data.

	Microsatellites (11 loci)							mtDNA							
Taxon/ population	N_μsat_	H_E_ (sd)	H_O_ (sd)	A_a_ (sd)	A_R_	A_P_	F_IS_	N_mt_	*k*	H_P_	*h*	π	*F*_S_	*D*	*g* (sd)
*F*. *p*. *pealei*	68	0.542 (0.064)	0.468 (0.019)	5.27 (2.80)	5.07	7	-	55	8	1	0.386	0.0018	-	-	-
NPAC	11	0.512 (0.085)	0.483 (0.046)	3.82 (1.66)	3.42	1	0.059	9	2	0	0.389	0.0006	0.477	0.156	4363 (2214)
SCCOA	13	0.574 (0.058)	0.552 (0.044)	4.00 (2.00)	3.12	0	0.040	12	3	0	0.621	0.0038	2.495	2.155	-243 (258)
ANDR	16	0.457 (0.078)	0.444 (0.039)	3.09 (1.45)	2.85	0	0.029	10	1	0	0.000	0.0000	-	0.000	-
RAT	17	0.503 (0.065)	0.449 (0.037)	3.73 (1.49)	3.09	1	0.110	15	4	2	0.371	0.0013	**-0.877**	**-1.660**	156 (275)
NEAR	4	0.507 (0.093)	0.525 (0.082)	2.40 (0.97)	-	0	-0.024	4	1	0	0.000	0.0000	-	0.000	-
COMM	7	0.515 (0.078)	0.416 (0.056)	3.36 (1.91)	3.40	2	0.205	5	2	0	0.400	0.0004	1.040	**0.973**	263 (673)
SJI	15	0.547 (0.075)	0.539 (0.039)	3.55 (1.97)	3.21	0	0.014	15	5	1	0.733	0.0022	**-0.951**	0.070	2689 (838)
*F*. *p*. *anatum*	49	0.517 (0.084)	0.474 (0.022)	5.09 (3.02)	5.05	3	-	45	5	0	0.468	0.0010	-	-	-
YUK	17	0.526 (0.084)	0.486 (0.037)	4.55 (2.50)	3.28	2	0.077	15	3	0	0.362	0.0007	**-0.918**	**-1.002**	**4413** (2482)
TAN	9	0.457 (0.102)	0.434 (0.500)	3.55 (2.16)	3.30	0	0.052	9	3	0	0.416	0.0007	**-0.081**	**-1.362**	**10000** (2976)
POR	9	0.466 (0.085)	0.414 (0.049)	3.55 (2.07)	3.10	1	0.118	7	1	0	0.000	0.0000	-	0.000	-
MCV	8	0.521 (0.097)	0.481 (0.054)	3.36 (1.69)	3.25	0	0.083	8	2	0	0.250	0.0005	-0.182	-1.054	10000 (7981)
LKAT	6	0.552 (0.062)	0.576 (0.061)	3.09 (1.14)	3.13	0	-0.047	6	3	0	0.733	0.0017	-0.304	0.311	1826 (1340)
*F*. *p*. *tundrius*	54	0.515 (0.084)	0.477 (0.021)	5.00 (2.97)	4.93	4	-	54	8	5	0.507	0.0010	-	-	-
HB	18	0.483 (0.087)	0.470 (0.037)	4.36 (2.06)	3.34	1	0.039	13	3	0	0.410	0.0007	**-0.790**	**-0.909**	**7662** (1714)
COL	36	0.514 (0.081)	0.481 (0.025)	4.64 (2.73)	3.23	2	0.065	41	7	4	0.543	0.0013	**-3.311**	**-0.466**	**5069** (489)
TOTAL/MEAN	186	0.558 (0.084)	0.472 (0.012)	6.36 (3.50)	3.21	10	0.060	169	15	8	0.492	0.0015	-	-	**-**

N_μsat_ = the number of samples for which microsatellite data were collected, H_E_ = expected heterozygosity, H_O_ = observed heterozygosity, A_a_ = average number of alleles per locus, A_R_ = allelic richness [76]; A_P_ = number of private alleles, F_IS_ = inbreeding coefficient, N_mt_ = the number of samples for which sequence data were collected, *k* = the number of haplotypes, H_P_ = the number of private haplotypes, *h* = haplotype diversity, π = nucleotide diversity; *F*_S_ = Fu’s *F*_S_ [52]; *D* = Tajima’s *D* [53], *g* = growth [74]. Statistically significant values are shown in bold. Overall values for subspecies (pooled from component populations) are provided in the shaded rows.

Overall, significant differentiation at microsatellite loci was observed across traditional metrics (F_*ST*_ = 0.101, *P* < 0.001; R_*ST*_ = 0.073, *P* < 0.003; χ2 = infinity, df = 22, *P* < 0.001). All populations comprising *F*. *p*. *pealei* were significantly differentiated from those comprising both *F*. *p*. *anatum* and *F*. *p*. *tundrius* (S1 Table). However, only 8 among 42 possible pairwise comparisons among and within populations representing *F*. *p*. *tundrius* and *anatum* were significant, and among these, significant differentiation between populations representing *F*. *p*. *tundrius* and *anatum* involved HB and LKAT (S1 Table). By contrast, population pairwise differentiation within and among populations from across the range of *F*. *p*. *pealei* was observed; all but 5 pairwise comparisons across analyses (all involving island groups of the Aleutian Island segment or Commander Islands and one involving SJI) were significantly different from zero (S1 Table). SJI was significantly differentiated from all other populations except the single instance of a non-significant F_*ST*_ value (SJI and NEAR).

**Fig 2:**
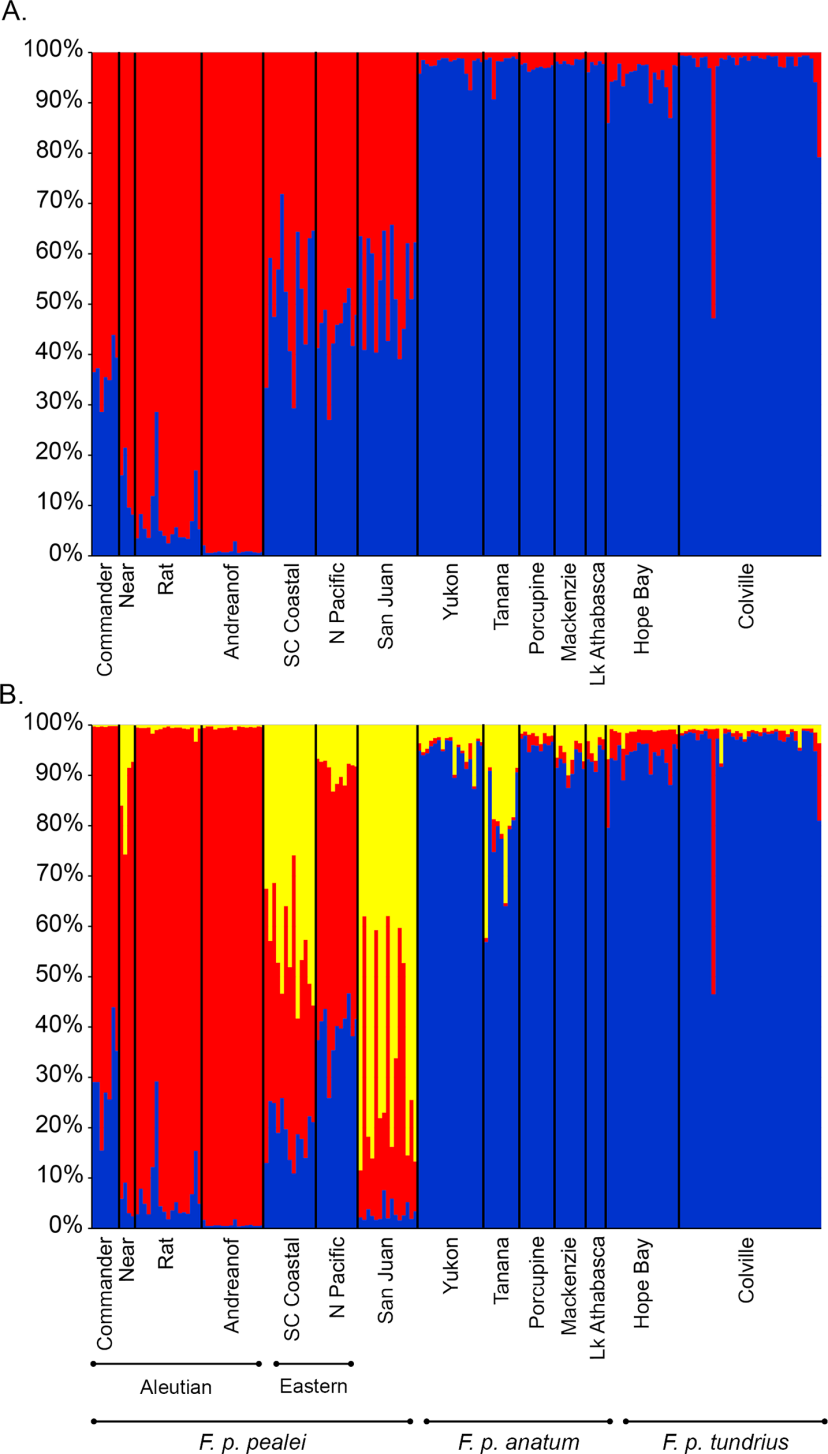
Genetic structure among Alaskan and western Canadian peregrine falcon populations, estimated by Bayesian clustering using STRUCTURE, without prior population information. A) most likely model (K = 2) estimated by maximizing ΔK (ΔK = 757.8, LnPr(X|K) = –4301.2); B) most likely model (K = 3), estimated by maximizing LnPr(X|K) (ΔK = 5.3, LnPr(X|K) = –4286.5).

Results of Bayesian cluster analyses using STRUCTURE 2.1 [61,62] indicated the two most likely models assigning breeding individuals was 2 (ΔK = 757.8, LnPr(X|K) = –4301.2) or 3 (ΔK = 5.3, LnPr(X|K) = –4286.5) based on maximizing the ΔK or LnPr(X|K) values, respectively (Fig 2). The 2-cluster model (Fig 2A) reflected 2 distinct clusters corresponding largely to individuals sampled from locales within the range *F*. *p*. *anatum/tundrius*, and within *F*. *p*. *pealei*. The 3-cluster model (Fig 2B) similarly clusters *F*.*p*. *anatum/tundrius* away from *F*. *p*. *pealei*, but in addition clustered the Aleutian/Commander islands group away from more easterly distributed populations within *F*. *p*. *pealei*; the latter showed substantial admixture with *F*. *p*. *anatum/tundrius* populations.

Variance in the distribution of microsatellite alleles was not significantly partitioned at the regional level in hierarchical AMOVA analyses based on the current subspecies hypotheses (Model A, Table 2). The greatest regional differentiation was uncovered when populations representing *F*. *p*. *tundrius* and *anatum* were pooled into a single group, and *F*. *p*. *pealei* was partitioned into the eastern (including SJI) and western segments (F_CTnuc_ = 0.093, *P* < 0.001; see Model D, Table 2). The C_SE_ tree generated from distances among all populations showed few nodes with bootstrap support greater than 50% (Panel A in S1 Fig). However, the C_SE_ tree showed strong bootstrap support (100%) between nodes separating the Aleutian and southeast and southcentral populations comprising *F*. *p*. *pealei*, and pooled populations comprising of *F p*. *anatum* and *tundrius* (Panel B in S1 Fig).

**Table 2:** Hierarchical analyses of variance for hypothesized groupings, based on data from 11 microsatellite loci (top) and mtDNA sequence data (bottom). Shown are fixation indices and percentage of the total variance explained by the hypothesized regional grouping and significance. The first grouping (Model A) tests the hypothesis that genetic variation is partitioned along currently accepted subspecies designations (e.g., *F*. *p*. *tundrius*, *F*. *p*. *anatum*, and *F*. *p*. *pealei*, with SJI placed with *pealei*). Bold values are significantly different from zero (*P* < 0.05 for mtDNA data; *P* < 0.0045 for microsatellite data).

		Variance Components					
Model	Hypothesized Groupings	F_IT_	F_IS_	F_SC_	F_CT_	% among groups	*P*_(among group)_
	**Microsatellite Loci**						
A	[COL,HB] [TAN,YUK,POR,LKAT,MCV] [SJI, NPAC,SCCOA, ANDR,RAT,NEAR,COMM]	**0.144**	0.038	**0.060**	0.059	5.89	0.005
B	[COL,HB] [TAN,YUK,POR,LKAT,MCV, SJI] [NPAC,SCCOA, ANDR,RAT,NEAR,COMM]	**0.141**	0.032	**0.063**	0.053	5.27	0.018
C	[COL,HB] [TAN,YUK,POR,LKAT,MCV] [SJI,NPAC,SCCOA] [ANDR,RAT, NEAR,COMM]	**0.140**	0.032	**0.042**	**0.072**	7.23	<0.001
D	[COL,HB,TAN,YUK, POR,LKAT,MCV] [SJI,NPAC,SCCOA] [ANDR RAT,NEAR,COMM]	**0.157**	0.032	**0.039**	**0.093**	9.35	<0.001
E	[COL,TAN,YUK,POR] [SJI,NPAC,SCCOA] [ANDR,RAT, NEAR,COMM] [MCV,LKAT, HB]	**0.142**	0.032	**0.041**	0.076	7.57	<0.001
F	[COL,TAN,YUK,POR,LKAT,MCV,HB] [SJI,NPAC,SCCOA,ANDR RAT,NEAR,COMM]	**0.162**	0.032	**0.055**	**0.084**	8.42	<0.001
	**MtDNA Control Region**						
A	[COL,HB] [TAN,YUK,POR,LKAT,MCV] [SJI,NPAC,SCCOA,ANDR,RAT,NEAR,COMM]	**-**	**0.130**	**0.169**	0.045	4.46	0.161
B	[COL,HB] [TAN,YUK,POR,LKAT,MCV, SJI] [NPAC,SCCOA, ANDR,RAT,NEAR,COMM]	**-**	**0.167**	**0.157**	**-**0.012	-1.22	0.495
C	[COL,HB] [TAN,YUK, POR,LKAT,MCV] [SJI,NPAC,SCCOA] [ANDR,RAT,NEAR,COMM]	**-**	**0.077**	**0.174**	**0.105**	**10.55**	0.019
D	[COL, HB, TAN, YUK, POR,LKAT,MCV] [SJI, NPAC, SCCOA] [ANDR, RAT, NEAR, COMM]	**-**	**0.074**	**0.200**	**0.136**	**13.63**	<0.004
E	[COL,TAN,YUK,POR] [SJI,NPAC,SCCOA] [ANDR,RAT, NEAR,COMM] [MCV,LKAT, HB]	**-**	**0.080**	**0.178**	**0.105**	**10.54**	0.012
F	[COL,TAN,YUK,POR,LKAT, MCV,HB] [SJI,NPAC,SCCOA,ANDR,RAT NEAR,COMM]	**-**	**0.120**	**0.189**	0.078	**7.85**	0.016

#### MtDNA data

**Fig 3:**
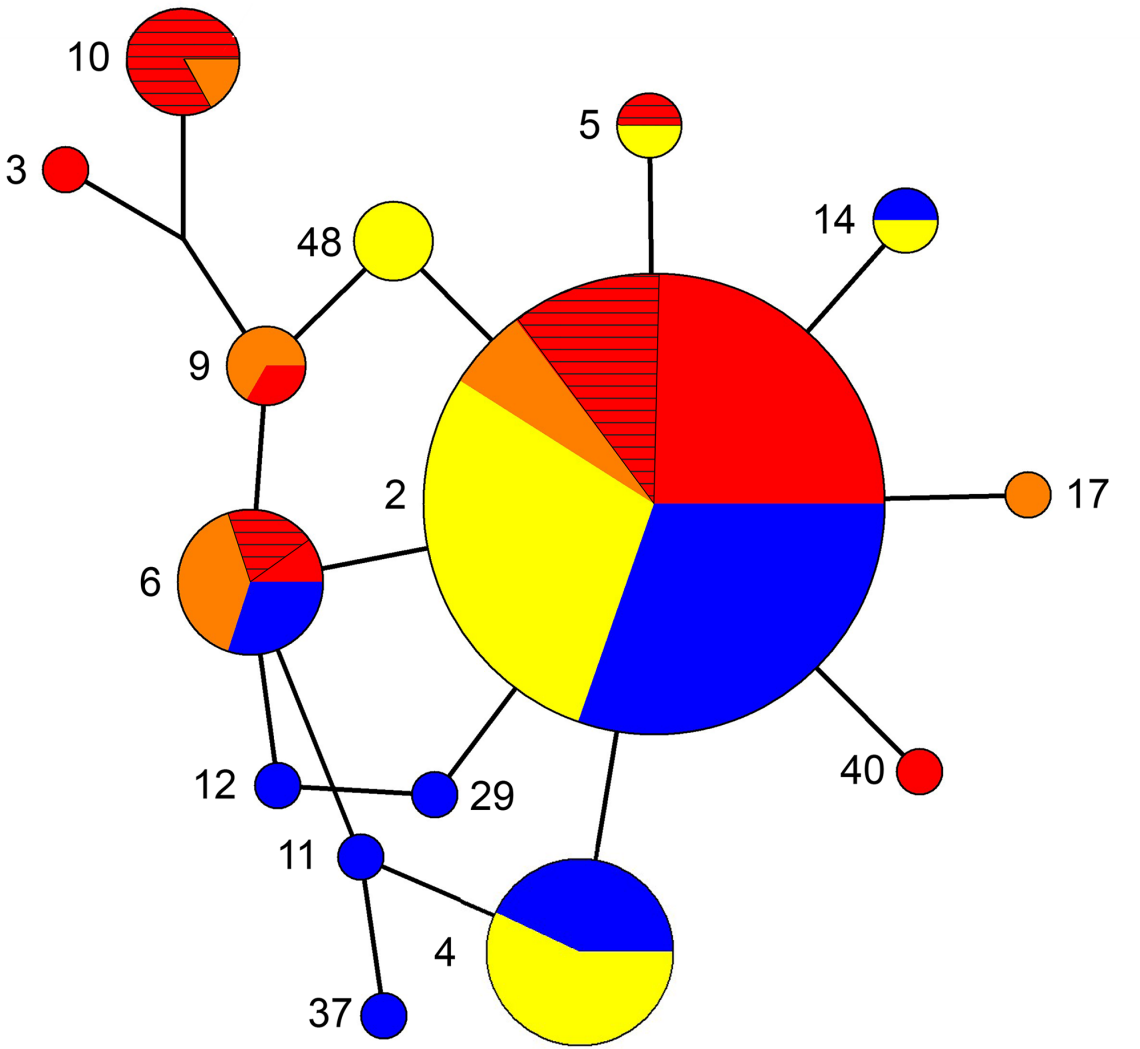
Unrooted reduced median network illustrating relationships among 15 mtDNA control region haplotypes observed among 169 peregrine falcons assayed from northern North American populations. *F*. *p*. *tundrius* populations are indicated in blue, *F*. *p*. *anatum* in yellow, *F*. *p*. *pealei* in red (hatched red for NPAC, SOLID red for the Aleutian Island locales), and the population on SJI in orange. The size of the circle node corresponds to the frequency of each haplotype.

We collected control region sequence data for 169 individuals from presumptive populations of *F*. *p*. *tundrius*, *anatum*, *pealei*, and SJI and observed 15 haplotypes [42]. Haplotype CR2 occurred in the highest frequency within almost all populations and representing 72% of haplotypes overall (Fig 3, S2 Table). Of the remaining 14 haplotypes, 2 were unique to *F*. *p*. *pealei*, 4 to *tundrius*, and 1 to SJI (S2 Table). Haplotype diversity was lower in *F*. *p*. *pealei* than in either *tundrius* or *anatum* (Table 1). Nucleotide diversity was higher in *F*. *p*. *pealei*. Near and Andreanof island groups (*F*. *p*. *pealei*) and Porcupine River (*F*. *p*. *anatum*) were characterized by a single haplotype, resulting in haplotype and nucleotide diversity values of zero (Table 1). We observed no significantly positive values of *D* or *F*_S_ in any population, suggesting there is little deviation from selective neutrality in mtDNA (see S1 Methods), save in one population: COMM showed a significantly positive value of *D* (*P* < 0.05; Table 1).

Although fewer significant pairwise population comparisons (based on either χ2 or ϕ_*ST*_) were observed for the mtDNA relative to the microsatellite data, we nevertheless observed a greater percentage of significant pairwise population comparisons between populations representing *F*. *p*. *pealei* and populations representing *F*. *p*. *anatum* and *tundrius*, versus within (S3 Table). LKAT was involved in the majority of significant comparisons within *F*. *p*. *anatum*, and SCCOA was involved in the majority of significant comparisons associated with *F*. *p*. *pealei* (excluding SJI, which was also strongly differentiated from most other populations, regardless of subspecies membership). Similar to microsatellite data, variance in the distribution of haplotypes was not significantly partitioned at the regional level in hierarchical AMOVA analyses based on the current subspecies hypotheses (Model A, Table 2), and similar to analyses based on microsatellite data, the greatest regional differentiation was uncovered when populations representing *F*. *p*. *tundrius* and *anatum* were pooled into a single region, and *F*. *p*. *pealei* was partitioned into the eastern and western segments (F_CTmit_ = 0.136, *P* < 0.004; see Model D, Table 2).

#### Estimation of levels and polarity in gene flow

M and θ values along with confidence limits estimated in MIGRATE from microsatellite genotypes and mtDNA were used to calculate N_e_m and N_f_m (θ*M), respectively, and suggested levels of asymmetry in gene flow varied among populations (Table 3, Table 4); confidence intervals around estimates of effective number of female migrants were relatively wide (Table 4). Among Alaskan regions, Arctic and interior boreal populations receive the most immigrants, based on microsatellite data (Table 3) and mtDNA (Table 4), respectively. Results for both marker types suggest that, on average, the maritime Alaskan peregrine (*F*. *p*. *pealei*) populations (Aleutian, SCCOA, NPAC) receive the fewest immigrants relative to the other Alaskan population segments assayed. Gene flow estimates based on the microsatellite data suggest that the western segment of *F*. *p*. *pealei* (Aleutians) receives, on average, the lowest immigration levels, generally <1 per generation per pairwise population comparison among all populations assayed (Table 3). The highest levels of gene flow (based on N_e_m) into the Aleutians originated from the North Pacific populations (NPAC), and effective female gene flow estimates (N_f_m) also suggest substantial gene flow originating from populations in the eastern portion of the distribution of *F*. *p*. *pealei* (SCCOA, NPAC), though confidence limits overlap (Table 4). Analyses of microsatellite data suggest there is asymmetrical gene flow (N_e_m) from SJI into populations within the range of all three subspecies (*F*. *p*. *pealei*: NPAC; *F*. *p*. *anatum*: LKAT; and *F*. *p*. *tundrius*: Arctic Alaska and Canada; see Table 3). Analyses of mtDNA data also suggest there is asymmetrical female gene flow (N_f_m) from SJI into interior Alaska (F. p. anatum; see Table 4). Average evolutionary dispersal (gene flow) between unaugmented interior Alaskan populations and Arctic Alaska, typically ascribed to *F*. *p*. *anatum* and *F*. *p*. *tundrius*, respectively, has been largely symmetrical (based on microsatellite loci, N_e_m, Table 3) or biased toward Arctic Alaska (based on mtDNA, N_f_m, Table 4). Interior Alaskan populations receive evolutionary dispersal from the western (Aleutian) segment of *F*. *p*. *pealei* (based on mtDNA, N_f_m, only; see Table 4). Signals of significant asymmetry in gene flow between augmented Canadian populations (represented in this analysis by LKAT and Arctic Canada [HB and MCV]) and unaugmented Alaskan populations have been from Arctic Canada into Arctic Alaska, as represented by COL (based on microsatellite loci, N_e_m, Table 3), and from interior Alaska into Arctic Canada (based on mtDNA, N_f_m, Table 4). Although estimates of effective female gene flow between Arctic Canada and Arctic Alaska are high and confidence intervals overlap, net gene flow is largely from Arctic Alaska into Arctic Canada (Table 4).

**Table 3:** Results of full gene flow model (all parameters allowed to vary independently) illustrating polarity and rates of evolutionary dispersal calculated from 11 microsatellite loci. Effective number of migrants per generation (N_e_m) and 95% confidence intervals are listed for each population pair in parentheses, where the columns are the population of origin and the rows are the population destination. Comparisons in bold text indicate the dominant direction of asymmetrical gene flow between population pairs with non-overlapping 95% confidence intervals. For example, the full model estimated asymmetrical gene flow between the SJI and NPAC, with significantly more gene flow from SJI into NPAC (N_e_m = 3.0 [CI = 2.2–4.0]) than from NPAC into SJI (N_e_m = 1.2 [CI = 0.8–1.6]). Values in gray cells represent comparisons with overlapping 95% confidence intervals. Total immigration for each population is shown in the right-most column, and emigration in the bottom row. Total immigration and emigration rates were calculated by totaling mean gene flow values to and from each individual population. Interior Alaska = YUK, POR and TAN, pooled; Aleutians = ANDR, RAT, NEAR and COMM, pooled; Arctic Canada = HB and MCV, pooled; Arctic Alaska = COL.

	Origin								
	[*F*. *p*. *pealei*]				[*F*. *p*. *anatum*]		[*F*. *p*. *tundrius*]		
Destination	Aleutians	SCCOA	NPAC	SJI	Interior Alaska	LKAT	Arctic Canada	Arctic Alaska	Total Immigration
Aleutians	-	0.3	1.4	0.8	0.4	0.9	0.6	0.6	5.0
		(0.2–0.5)	(1.1–1.8)	(0.6–1.0)	(0.2–0.5)	(0.7–1.2)	(0.5–0.9)	(0.4–0.9)	
SCCOA	**1.2**	-	0.9	2.2	1.1	1.3	1.7	1.0	9.4
	**(0.8–1.7)**		(0.6–1.3)	(1.7–3.0)	(0.8–1.6)	(0.9–1.8)	(1.2–2.3)	(0.7–1.4)	
NPAC	**2.6**	1.2	-	**3.0**	**1.5**	**2.6**	1.6	1.8	14.3
	**(1.9–3.5)**	(0.8–2.0)		**(2.2–4.0)**	**(1.0–2.2)**	**(1.9–3.6)**	(1.1–2.3)	(1.3–2.6)	
SJI	**2.3**	1.3	1.2	-	0.8	0.5	0.3	0.9	7.3
	**(1.8–2.9)**	(0.9–1.7)	(0.8–1.6)		(0.6–1.1)	(0.4–0.8)	(0.2–0.5)	(0.5–1.2)	
Interior Alaska	**0.8**	1.7	0.5	0.8	-	2.3	2.8	3.5	12.4
	**(0.5–1.1)**	(1.3–2.2)	(0.3–0.8)	(0.6–1.1)		(1.8–2.9)	(2.2–3.4)	(2.8–4.2)	
LKAT	1.4	2.4	1.1	**1.6**	3.0	-	1.3	1.9	12.7
	(0.9–2.0)	(1.7–3.7)	(0.7–1.7)	**(1.2–2.1)**	(2.2–4.1)		(0.9–1.8)	(1.3–2.7)	
Arctic Canada	1.0	0.9	1.1	**1.6**	2.0	1.8	-	1.2	9.6
	(0.7–1.3)	(0.6–1.3)	(0.8–1.5)	**(1.1–2.9)**	(1.5–2.6)	(1.4–2.3)		(0.9–1.6)	
Arctic Alaska	1.1	**1.9**	2.1	**3.7**	2.7	1.9	**2.4**	-	15.8
	(0.8–1.5)	**(1.5–2.5)**	(1.7–2.7)	**(3.0–4.5)**	(2.1–3.3)	(1.5–2.4)	**(1.9–3.0)**		
Total Emigration	10.4	9.7	8.3	13.7	11.5	11.3	10.7	10.9	-

**Table 4:** Results of full gene flow model (all parameters allowed to vary independently) illustrating polarity and rates of evolutionary dispersal calculated from mtDNA control region data. Effective number of female migrants per generation (N_f_m) and 95% confidence intervals are listed for each population pair in parentheses, where the columns are the population of origin and the rows are the population destination. Comparisons in bold text indicate the dominant direction of asymmetrical gene flow between population pairs with non-overlapping 95% confidence intervals. For example, the full model estimated asymmetrical gene flow between the SJI and NPAC, with significantly more gene flow from Interior Alaska into SCCOA(N_f_m = 6.3 [CI = 1.7–23.6]) than from SCCOA into Interior Alaska (N_f_m = 0.2 [CI = 0.0–0.9]). Values in gray cells represent comparisons with overlapping 95% confidence intervals. For example, the full model estimated asymmetrical gene flow between the SJI and NPAC, with significantly more gene flow from Interior Alaska into SCCOA(N_f_m = 6.3 [CI = 1.7–23.6]) than from SCCOA into Interior Alaska (N_f_m = 0.2 [CI = 0.0–0.9]). Total immigration for each population is shown in the right-most column, and emigration in the bottom row. Total immigration and emigration rates were calculated by totaling mean gene flow values to and from each individual population. Interior Alaska = YUK, POR and TAN, pooled; Aleutians = ANDR, RAT, NEAR and COMM, pooled; Arctic Canada = HB and MCV, pooled; Arctic Alaska = COL.

	Origin								
	[*F*. *p*. *pealei*]				[*F*. *p*. *anatum*]		[*F*. *p*. *tundrius*]		
Destination	Aleutians	SCCOA	NPAC	SJI	Interior Alaska	LKAT	Arctic Canada	Arctic Alaska	Total Immigration
Aleutians	-	34.4	17.2	2.5	0.4	0.0	5.7	0.0	60.2
		(7.1–240.1)	(2.6–269.1)	(0.2–27.9)	(0.1–1.3)	(0.0–3.0)	(0.2–196.4)	(0.0–6.0)	
SCCOA	7.5	-	0.0	14.9	**6.3**	0.5	5.7	4.4	39.3
	(0.8–61.6)		(0.0–24.5)	(3.4–97.9)	**(1.7–23.6)**	(0.0–8.2)	(0.2–196.4)	(0.6–23.5)	
NPAC	3.8	3.8	-	0.0	1.3	0.5	22.8	4.4	36.6
	(0.2–42.3)	(0.2–58.9)		(0.0–7.1)	(0.5–2.6)	(0.0–8.2)	(2.4–444.1)	(0.6–23.5)	
SJI	0.0	11.5	7.7	-	0.4	0.5	0.0	6.6	26.7
	(0.0–15.7)	(1.4–109.9)	(0.8–149.2)		(0.1–1.3)	(0.0–8.2)	(0.0–72.8)	(1.2–30.2)	
Interior Alaska	**157.9**	0.2	3.8	**8.7**	-	0.6	4.8	0.0	176.0
	**(53.3–622)**	(0.0–0.9)	(0.2–95.9)	**(2.9–64.6)**	-	(0.2–1.6)	(2.9–7.7)	(0.0–0.3)	
LKAT	7.5	11.5	1.9	1.2	3.2	-	2.1	2.1	29.5
	(0.8–61.6)	(1.4–109.9)	(0.1–66.0)	(0.0–19.2)	(0.6–24.7)		(0.3–18.6)	(0.3–18.6)	
Arctic Canada	0.0	19.1	1.9	3.7	**267.6**	5.7	-	17.1	315.1
	(0.0–15.7)	(3.1–155.4)	(0.1–66.0)	(0.5–35.8)	**(57.2–3187.4)**	(0.2–196.4)		(1.5–366.8)	
Arctic Alaska	7.5	3.8	11.5	3.7	**17.7**	4.4	2.2	-	50.8
	(0.8–61.6)	(0.2–58.9)	(1.5–198.6)	(0.5–35.8)	**(7.2–43.6)**	(0.6–23.5)	(0.1–16.2)		-
Total Emigration	184.2	84.3	44.0	34.7	296.9	12.2	43.3	34.6	

#### Genetic signals of population demography

There was evidence of significant heterozygosity excess, expected in the case of a recent bottleneck, only in SJI (*P* < 0.001) and only under the IAM. Analysis under the SMM and TPM also gave evidence of significant heterozygosity deficit expected in the case of recent population growth due to the influx of alleles, in Alaskan *F*. *p*. *anatum* (*P* < 0.003), represented by pooling data from POR, YUK, and TAN. After the application of Bonferroni correction, no significant heterozygosity excess or deficit was observed in falcons sampled from Canada (MCV and LKAT, *P* > 0.006; HB, *P* > 0.008), Arctic Alaska (COL, *P* > 0.100), or the eastern or Aleutian components of *F*. *p*. *pealei* (*P* > 0.006, *P* > 0.016, respectively).

Significant fluctuations in historical population demography, inferred from tests of neutrality of the mtDNA data (Table 1), were observed in RAT, YUK, TAN, HB, and COL, *viz* significantly negative Fu’s *F*_S_ (*P* < 0.02) and Tajima’s *D* (*P* < 0.05). SJI also showed a significantly negative Fu’s *F*_S_. FLUCTUATE analyses of mtDNA sequence (Table 1) uncovered a signal of population increase in two locales within the distribution of *F*. *p*. *tundrius* (COL, HB) and in peregrines in two locales within the range of *anatum* in Alaska (YUK and TAN).

## Discussion

### Genetic diversity, differentiation, demography, and gene flow

Although inference of relationships among North American subspecies made by Brown et al. [7] were based on neutral genetic data from populations that were augmented by introduction of peregrines with ancestry from exotic locales, our analyses from non-augmented populations in Alaska corroborate their finding, as well as that of White et al. [6] that there is no discernable regional-level genetic differentiation between *F*. *p*. *anatum* and *tundrius* in high latitude habitats in western North America. A close relationship among populations representing *F*. *p*. *anatum* and *tundrius* is supported by lack of regionally significant variance in allele and haplotype frequencies and estimates of substantial levels of gene flow between populations ascribed to one or the other subspecies, the high percentage of shared common haplotypes, and general lack of diagnostic genetic markers. These results highlight a signal of both historical and contemporary gene flow between *F*. *p*. *anatum* and *F*. *p*. *tundrius*, even in Alaskan populations, which were not augmented subsequent to the mid 20^th^ century population decline.

By contrast, and also like Brown et al. [7], we found that *F*. *p*. *pealei* is differentiated at the regional level from populations currently ascribed to the other two subspecies. Traditional population differentiation metrics based on both marker types showed significant structuring among most populations comprising *F*. *p*. *pealei* and those comprising the mainland and Arctic Alaskan and Canadian populations, supporting a distinction of peregrines within the distribution of *F*. *p*. *pealei*. As well, Bayesian analyses of population structure suggested peregrine falcons in populations belonging to *F*. *p*. *pealei* cluster away from populations ascribed to the other two subspecies.

A novel finding is that of substructuring within *F*. *p*. *pealei*; the Aleutian segment is genetically differentiated from the North Pacific segment (the Alexander and Haida Gwaii archipelagos) as well as from populations along south central coastal Alaska (Icy Bay, Kenai Fjords, Kodiak Island), for which subspecies attribution is unclear [77]. Peregrine falcons occupying the San Juan Islands of Washington are distinct from other populations occupying habitats ascribed to *F*. *p*. *pealei*, and from mainland Alaskan and Canadian populations currently ascribed to *F*. *p*. *anatum*; nevertheless, falcons occupying the San Juan Islands consistently cluster with *F*. *p*. *pealei* in AMOVA tests. Coastal Alaskan populations comprising the eastern arm of *F*. *p*. *pealei*, including locales along the northern Gulf of Alaska’s Lost Coast (SCCOA: Yakutat and Kenai Fjords) that were recently attributed to *F*. *p*. *anatum* based on morphology and migratory behavior [77], appear to include individuals with mixed ancestry, as does SJI. For SJI, this is possibly due to a trend toward female-biased gene flow from populations occupying Arctic Alaska, although confidence intervals for immigration and emigration overlap. For peregrines occupying the Lost Coast of Alaska (SCCOA), this may be due to female-biased gene flow from interior boreal Alaska populations. Clarification of local relationships for SJI will require additional sampling of temperate latitude peregrine falcons. Similarly, elucidation of local relationships of south central Alaskan maritime populations may emerge upon increased sampling. Given we have detected gene flow between potentially augmented Canadian populations and unaugmented Alaskan populations, and possible admixture in certain portions of the range of *F*. *p*. *pealei* (e.g., the Lost Coast of Alaska), we suggest a comprehensive survey of North American populations be conducted to better understand the relationships among contemporary North American peregrine falcons.

Surprisingly, despite the well-characterized DDT-induced decline of peregrine falcon populations in North America during the mid 20^th^ century, analyses using the approach of Cornuet and Luikart [68] found no evidence for a recent population bottleneck in peregrines occupying unaugmented habitats in Alaska, including peregrines sampled from boreal and Arctic locales within the distribution of the two subspecies (*F*. *p*. *anatum* and *tundrius*, respectively) that were listed for several decades as endangered or threatened under two Acts of the U. S. Congress. However, heterozygosity deficit, a genetic signal of demographic increase attributed to the influx of alleles [68], was observed in boreal (*F*. *p*. *anatum*) peregrines in Alaska. This is consistent with data emerging from long-term field surveys that have documented an increase in the number of occupied territories along the upper Yukon River from 1970 to 2012 [27]. Although average generation times for western high latitude peregrine falcons are not known, given an average generation time estimate of 6.8 years for this species [78], the increase on the Yukon River would have occurred across approximately 6 generations. We note also that falcons occupying interior boreal and Arctic regions of Alaska show growth signals (*g*) consistent with population expansion over more historical time periods (Table 1). It is unclear why the analysis using BOTTLENECK failed to uncover a genetic signature (i.e., heterozygosity excess) of recent population fluctuation in Colville River peregrines, which, like peregrine nesting along the Yukon River, declined in the mid-1950s, then recovered over a 40-year period [27]. Hailer et al. [79] suggest that species with long generation times, such as raptors, may be buffered from rapid loss of genetic diversity when their populations pass through a bottleneck. As well, based on surveys conducted along the Colville River between 1981 and 2002, the number of occupied nest sites and cliffs by nesting peregrine falcons was less than 30 for only a few years, increasing to around 50 nest sites by 1988 [32]. This suggests a severe bottleneck (just over 25 effective breeders) that nevertheless 1) was possibly of short duration, spanning only a few generations at most; 2) occurred only a few generations prior to the collection of the samples from the Colville population used in the BOTTLENECK analysis (mostly 2001–2002; see [42]), and 3) occurred in a species that is thought to have never been abundant in North America, even prior to the mid 20^th^ century decline [20,24]. Under those circumstances, the demographic bottleneck may have failed to generate a genetic signature that can be detected using the methods employed by BOTTLENECK [68].

However, we note that in a population that has undergone a bottleneck, we would expect to see certain signatures in the mtDNA, including low nucleotide diversity coupled with high haplotype diversity, negative Tajima’s *D*, and negative Fu’s *F*_S_ [80,81]. Haplotype diversity at the mtDNA control region for the peregrines assayed here is generally low, with only three of the 14 populations with haplotype diversity values greater than 0.50 (Table 1). However, certain populations, including those representing interior boreal (YUK, TAN) and Arctic Alaskan (COL) peregrines, demonstrated significantly negative *F*_S_ and *D* coupled with low nucleotide diversity but also signatures of growth (*g*) (Table 1). Combined, these metrics provide a signal, albeit weak, of population fluctuations, including possibly population bottlenecks. We acknowledge, however, the difficulty in parsing the roles of selection and population demography as inferred from widely-used neutrality metrics, including *F*_S_ and *D* [80,81].

Although we uncovered moderate population-level structuring overall, gene flow rate estimates among subspecies and populations, based on both marker types, suggest substantial gene flow within and among regions. The Aleutian segment of *F*. *p*. *pealei* is notable for its isolation: although they provide immigrants into the eastern segment of *pealei*, across generations and, on average, Aleutian Island populations appear to receive the fewest effective immigrants (N_e_m) among assayed populations. Augmentation of Aleutian populations appears to be largely via female gene flow from other southcentral and southeast Alaskan maritime populations. Other populations of *F*. *p*. *pealei* occupying habitats along the coast of Alaska, British Columbia, and Washington are connected via varying levels of evolutionary dispersal with boreal and Arctic Canadian and Alaskan populations. In particular, the population on the San Juan Islands represents a source for evolutionary dispersal into interior boreal and Arctic Canada and Alaska.

Of considerable interest is the finding of gene flow between the Canadian populations, presumably augmented, and the non-augmented populations in interior boreal and Arctic Alaska, including estimates of asymmetrical gene flow (N_e_m) from Arctic Canada into Arctic Alaska, based on microsatellite data. However, significant signals of asymmetry in gene flow between augmented Canadian populations and non-augmented Alaskan populations have been in the form of effective female gene flow (N_f_m) from interior boreal Alaska into Arctic Canada. We note that the female peregrines typically disperse farther than males from natal areas to breed [19], suggesting female bias in gene flow. Estimates of effective female gene flow between Arctic Canada and Arctic Alaska are high, but with overlapping confidence intervals; still, net gene flow appears to be out of the Arctic Alaska, suggesting that boreal and Arctic Alaska have represented a source of dispersing females for Arctic Canada populations. We caution that gene flow values provide a rough estimation of the rate of gene exchange among population groupings, and should not be viewed as precise measures, but rather within the context of whether certain regional groupings within northwestern North American peregrine falcons are substantially isolated from other groupings, as observed elsewhere [7,46].

The three recognized subspecies of *F*. *peregrinus* examined here displayed similar levels of genetic diversity overall, although *F*. *p*. *pealei* showed lower levels of mtDNA haplotype diversity, driven largely by low number of haplotypes in the Aleutian segment of the subspecies. Low genetic diversity cannot be attributed to a small population size; population size of peregrines in the Aleutian Islands is thought to be similar to elsewhere within the range of *F*. *p*. *pealei* [82], and the population size of peregrines within the range of *pealei* is higher than found within the geographic range of either *anatum* or *tundrius*. Although Murie [83] estimated that 100 breeding pairs occupied the Aleutian Island chain, densities in the Rat and Near island groups and Buldir [84] and the eastern portion of the Aleutian Archipelago [85] led Ambrose et al. [82] to estimate that approximately 300 pairs occupied the Aleutians. Based on extrapolation from extensive surveys on the Rat Island group [86], more recent estimates of abundance across the North American portion of the Aleutian chain range from 375–580 breeding pairs [87]. The region between the Kenai Peninsula to southeast Alaska is estimated to hold more than 140 territories, and White [84] tallied 69 eyries along the Alaska Peninsula and on Kodiak Island. An axiom in conservation biology is that island populations are more prone to extinction than mainland populations [88–91], and island populations often demonstrate lower levels of genetic diversity and increased inbreeding [92]. Insular populations of peregrine falcons on oceanic island archipelagos elsewhere are characterized by lower levels of genetic diversity than mainland populations, and in at least one case these low levels of variability may have fostered inbreeding depression [47]. While our results showed that average levels of genetic diversity in populations in the Aleutian Island archipelago are lower than elsewhere, a significantly positive inbreeding coefficient was not uncovered. Lower levels of genetic variability in the Aleutian distribution of *F*. *p*. *pealei* are likely facilitated, at least in part, by the isolation of the Aleutian Island archipelago and restricted augmentation via gene flow from mainland populations and attendant *relative* effective population size.

Our analyses, similar to those of Brown et al. [7], fail to support the current subspecies designations as applied to peregrine falcons occupying high latitude North America, particularly with regard to falcons sampled from within the distribution currently ascribed to either *tundrius* or *anatum*. The concept of ‘subspecies’ remains controversial, particularly within ornithology [2,3,93–96]. Presumably, an evolutionary basis for taxonomy requires the application of genetic tools to questions involving subspecies designations. As pointed out by Brown et al. [7], however, genetic criteria for subspecies uniqueness are not universal and depend upon the investigator and metric used for subspecies diagnosis. For example, Zink [3] uses monophyly of mtDNA haplotypes as a criterion for attributing subspecies. Applied in this case, all peregrine falcons in the northwestern high latitudes of North America would belong to a single subspecies. Under both Barrowclough’s [94] criterion of ‘predictiveness,’ which posits that subspecies designations should allow the prediction of a suite of quantifiable character states, and Crandall et al.’s [97] criteria of ‘ecological and genetic exchangeability’, the eastern and western distributions of *F*. *p*. *pealei*, which are genetically and to some degree morphologically distinguishable [23], would each qualify as separate subspecies, whereas *F*. *p*. *anatum* and *tundrius* would be pooled into a single subspecies. We note that *F*. *p*. *pealei* in both segments (Aleutian and eastern) occupy island archipelagos (albeit one more isolated from mainland populations than the other two) and unlike the boreal and Arctic peregrines tend to show little to no migratory behavior, a characteristic that may contribute to greater population and regional structuring, as seen in other avian species [98,99] and particularly in insular populations [100,101].

## Conclusions

Our genetic survey provides insights into the status and delineation of natural populations as well as a better understanding of gene flow among unaugmented high latitude peregrine falcons. While we make no attempt at formal taxonomic revision, our data suggest that peregrine falcons occupying habitats in Alaska and the North Pacific coast of North America belong to two distinct regional groupings–a coastal grouping (*F*. *p*. *pealei*) and a boreal/Arctic grouping (currently *F*. *p*. *anatum* and *tundrius*), each comprised of discrete populations that are variously intra-regionally connected. Gene flow estimates indicating that distant high-latitude populations in Canada provide immigrants into and receive immigrants from mainland Alaskan populations ascribed to both previously listed subspecies. This suggests that management prescriptions applied at a distance may have unforeseen consequences in locally-managed populations of species with high gene flow potential, even in species characterized by reasonably strong philopatry. This highlights the importance of inter-regional and international cooperation in management and conservation in such species [102]. Differentiation among populations within the distribution of *F*. *p*. *pealei* is stronger than among boreal/Arctic populations, possibly due to decreased or absent migratory tendencies that restrict gene flow into the Aleutian Island segment of *F*. *p*. *pealei*. Thus, peregrines residing on islands in the Aleutian Archipelago likely remain the least genetically impacted by DDT-induced population fluctuations that fostered the mid 20^th^ century decline of peregrines in North America and subsequent augmentation by non-native subspecies.

## Supporting information

S1 Methods Supplementary material showing details of laboratory methods and data analyses. (DOCX)

S1 Fig Neighbor-joining network based on C_SE_ distances and 11 microsatellite loci. Illustrated are relationships among A) 14 populations of peregrine falcon in high latitudes habitats in Canada, Alaska and the Commander Islands, Russia; and B) three subspecies (*F*. *p*. *anatum*, *F*. *p*. *tundrius*, and *F*. *p*. *pealei*). Populations within the distribution of *F*. *p*. *pealei* are designated with red branches, within the distribution of *F*. *p*. *anatum* with yellow branches, and within the distribution *of F*. *p*. *tundrius* with blue branches. Values in nodes designate bootstrap values, based on 1000 permutations (only values > 50% are shown).
(TIF)

S1 Table Results of traditional population differentiation analyses, based on microsatellite fragment data. Shown are values for pairwise χ2 (above diagonal) and F_*ST*_ (below diagonal) comparisons among populations of *F*. *peregrinus* within Alaska, based on data from 11 microsatellite loci. See manuscript for locales associated with acronyms. Values in bold, and values listed as ∞ (= infinity) indicate significant differences in the distribution of alleles/haplotypes or variance in allelic/haplotypic frequency, respectively, following Bonferroni corrections (α = 0.0045). The population on the San Juan Islands (SJI) is considered a contact zone between continental populations (*F*. *p*. *anatum*) and maritime populations (*F*. *p*. *pealei*).
(DOCX)

S2 Table Distribution of mitochondrial DNA haplotypes among peregrine falcon populations in high latitude western North America. Populations augmented with non-native subspecies following the population decline are shaded.
(DOCX)

S3 Table Results of traditional population differentiation analyses, based on mitochondrial DNA control region sequences. Shown are values for pairwise χ2 (above diagonal) and ϕ_ST_ (below diagonal) comparisons among populations of *F*. *peregrinus* within Alaska, based on sequence data from the mitochondrial DNA control region. See manuscript for locales associated with acronyms. Values in bold or (for χ2) designated as **∞** (infinity) indicate significant differences in the distribution of haplotypes or variance in haplotype frequency (P < 0.05), respectively. The population on the San Juan Islands (SJI) is considered a contact zone between continental populations (*F*. *p*. *anatum*) and coastal populations (*F*. *p*. *pealei*).
(DOCX)
