# Supplementary material for: Intraspecific evolutionary relationships among peregrine falcons in western North American high latitudes
Source: PLoS One. 2017 Nov 17;12(11):e0188185. doi: 10.1371/journal.pone.0188185 (PMC5693296; doi:10.1371/journal.pone.0188185)
Supplement: S1 Methods — (DOCX) [file pone.0188185.s001.docx]

**S1 Methods**

**Laboratory techniques**

DNA from blood samples collected from POR, TAN, YUK, LKAT, HB, and MCV during 1985-1989 was extracted at Los Alamos National Laboratory, using standard phenol-chloroform extraction techniques [1]; extracts were forwarded to the U. S. Geological Survey Alaska Science Center (USGS-ASC) Molecular Ecology Laboratory for genotyping and sequencing. Blood and feather samples collected from COL (200-2012), the Aleutian Islands (RAT, ANDR, NEAR, COMM), NPAC, SCCOA, YUK (2006-2012), and SJI were extracted in the USGS-ASC using procedures outlined in Medrano et al. [2], modified as outlined in Sonsthagen et al. [3]. For feather and museum samples, extraction procedures were further modified as described in Talbot et al. [4]. Genomic DNA extractions were quantified using fluorometry and diluted to 50 ng/µL working solutions.

We genotyped each individual at 11 microsatellite loci known to be polymorphic in peregrine falcons [4–7] and sequenced a 559 base pair segment of domain 1 of the mtDNA control region, following procedures described in detail elsewhere [4,8]. Some feather and museum samples did not produce a product using the mtDNA primers listed in Talbot et al. [4], so we designed a set of internal primers (H299 5’–GCAGTAGTCCGAACCTCGTG–3’and L257 5’–ACCCACATTAGTTCACGTAG–3’) that amplified two smaller, overlapping portions of the target sequence. For quality control purposes, DNA from two to five individuals representing each designated population were extracted, amplified, and sequenced in duplicate.

**Data analyses**

An unrooted phylogenetic network for mtDNA control region was constructed from haplotypes in NETWORK 4.610 (Fluxus Technology, Clare, United Kingdom) using the reduced median method [9], to illustrate possible reticulations in the gene tree due to homoplasy.

**Genetic variability and tests of neutrality**

Exploratory analyses failed to detect linkage disequilibrium or deviations from Hardy-Weinberg equilibrium (HWE) when microsatellite data from samples collected from YUK peregrines during the 1980s and 2000s were pooled, suggesting they represented a single breeding population; we retained these samples in a single population (YUK). For microsatellite data, observed and expected heterozygosities and allelic frequencies were calculated for all populations in GENEPOP’007 [10]. Allelic richness [11], across populations and subspecies, and inbreeding coefficients (*F_IS_*) were calculated using FSTAT 2.9.3 [12]. Each locus in the proposed populations was tested for deviation from HWE in GENEPOP’007, using the Markov chain parameters provided (dememorization number = 1,000, number of batches = 100, and number of iterations per batch = 1,000). Since loci were not mapped, each pair of loci was tested for linkage disequilibrium in GENEPOP’007 using the same Markov chain parameters. To estimate genetic diversity metrics (haplotype diversity, *h*, and nucleotide diversity, π, and number of haplotypes) for mtDNA control region sequence data, we employed ARLEQUIN 3.1 [13]. We tested the hypothesis of selective neutrality for mtDNA control region sequence data, and for historical fluctuations in population demography, using Fu's *F*_S_ [14] and Tajima's *D* [15] implemented by ARLEQUIN. [14]. A negative value of *F*_S_ provides evidence for an excess number of alleles, expected in the case of a recent population expansion (or from genetic hitchhiking); a positive value, evidence for a deficiency of alleles, would be expected from overdominant selection or a recent population bottleneck (Fu 1997). Thus, significantly large negative *F*_S_ values can be interpreted as evidence of population expansion. Interpretation of Tajima’s *D*, a “general purpose” test [16] variously sensitive to both demographic and selection processes, is less straightforward. When *D* significantly greater than zero, it can be interpreted as resulting from balancing selection, or a recent population bottleneck. A significantly negative *D* can be interpreted as either population expansion or purifying selection; *D* will tend to be negative under positive selection in a population that has not undergone any demographic change [17]. Both values are widely used for interpreting patterns of nucleotide sequence variation, but Zeng et al. [16] suggest that Tajima’s may have low sensitivity to departures from neutrality. Fu’s *F*_S_ is generally considered a more sensitive indicator of population expansion and genetic hitchhiking than Tajima’s *D*. Because no test is powerful in every stage of the selection process [16], we provide information from both metrics. We applied critical significance values of 5%, which for Fu's *F*_S_ requires a *P*-value below 0.02 [14].

**Population structure and regional differentiation**

Global multilocus estimates of F*_ST_* [18] and R*_ST_* [19] based on microsatellite data were obtained using FSTAT; estimates of interpopulational variance in allele frequency were derived using ARLEQUIN. Significance of F*_ST_* and R*_ST_* values were based on random permutation tests (n = 1000), whereby alleles were randomly permuted between the two populations. Population differentiation based on the distributions of microsatellite alleles and genotypes across populations were examined using Fisher’s combined probability test [20,21] implemented by GENEPOP’007, with significance based on random permutation tests (n =1,000) across loci between populations. *P*-values for multilocus microsatellite comparisons of variance in allele frequency and distribution of alleles and genotypes were adjusted by applying Bonferroni corrections. Microsatellite data were also analyzed in STRUCTURE 2.1 [22] to detect the occurrence of population structure without *a priori* knowledge of putative populations. This program uses multilocus allelic frequencies to assign individuals probabilistically to populations. Data were analyzed using an admixture model, assuming correlated frequencies among populations, and results were generated from 500,000 Markov chain Monte Carlo iterations following a burn-in period of 50,000 iterations with range of possible populations (*K*) ranging from 1-10. This analysis was repeated 5 times for each value of *K* to ensure that results were consistent across runs, as suggested by Pritchard et al. [22]. We used the methods of Pritchard et al. [22] and Evanno et al. [23] to determine the most likely number of clusters.

We used the likelihood ratio test criterion in MODELTEST 3.06 [24] to determine the evolutionary distance model that best fit the mtDNA control region sequence data. These distances were used to calculate population pairwise F*_ST_* (ϕ_ST_; [25]) and were tested for significance using ARLEQUIN. Population differentiation based on the distributions of mtDNA haplotypes were examined using the log-likelihood (G) test [26] implemented by GENEPOP, with significance based on random permutations tests (n =1,000).

We used analyses of molecular variance (AMOVA) conducted using the neutral markers (mtDNA sequence and microsatellite genotype data) to test for significant geographic partitioning of *a priori* hypothesized regional units (subspecies), using ARLEQUIN. Calculations for mtDNA sequence data were performed incorporating a distance matrix based on the model of evolution determined by MODELTEST to best fit the data, and without weighting by a distance model. In addition to the *a priori* groups of testable subspecies hypotheses applicable to peregrine falcons of North America, we experimented with various *a posteriori* groups in AMOVA analyses for both marker types. The *a posteriori* groups were used to test the relationships of certain populations to named subspecies (for example, the placement of SCCOA and SJI relative to *anatum* and *pealei*) as well as to explore results of subsequent analyses (for example, microsatellite population trees, and Bayesian analyses of population structuring). We assumed that the best geographic subdivisions were significantly different from random distributions and had maximum among group variance (Φ_CT_ values). Thus, given concordance between the distribution of genetic subdivisions at mtDNA, and presumed subspecies delineations, values of Φ_CT_ should be larger than alternative groupings, and significant. To further examine regional relationships, we also constructed a network based on Cavalli-Sforza and Edwards [27] genetic distances (C_SE_) among microsatellite loci and neighbor-joining methods [28], using POPULATIONS 1.2.30 [29]. Populations were pooled into assigned subspecies, except for *F. p. pealei*, which was partitioned into the Aleutian group and the eastern group, based on results of population differentiation analyses. Networks were visualized in TREEVIEW 1.6.6 [30].

**Estimation of levels and polarity in gene flow**

To estimate gene exchange among subspecies, we used MIGRATE 3.0.3 [31,32] to calculate the number of migrants among populations per generation (4N_e_m) for nuclear microsatellites, and number of female migrants per generation (N_f_m) for mtDNA. The estimated parameters were θ (4N_e_ µ, where µ is the mutation rate), and M (m/µ); we note that the gene flow estimate is more commonly expressed as 4Nm, which is θ*M. Significance of asymmetry in gene flow was based on non-overlapping 95% confidence intervals generated when estimating full models, in which composite measures θ (4N_e_µ or N_f_µ) and M (m/µ) were estimated individually from the data and allowed to vary in symmetry between populations. MIGRATE inputs included maximum likelihood search parameters, ten short chains (1,000 used trees of 20,000 sampled), 5 long chains (10,000 used trees of 200,000 sampled) and 5 adaptively heated chains (start temperatures: 1, 1.5, 3, 6, and 12; swapping interval =1). Full models were run 5 times and parameter estimates converged. For this analysis, HB and MCV, which showed no significant interpopulational structuring, were pooled to represent Arctic Canada; similarly, POR, TAN and YUK, were pooled to represent interior Alaska and the different Aleutian Archipelago islands were pooled to represent the Aleutian Islands as a single group.

**Genetic signals of changes in population demography**

Genetic evidence for fluctuations in recent demography was evaluated for the microsatellite loci using Bottleneck 1.2.02 [33]; evidence of more historical population changes was evaluated using mtDNA sequence data, employing FLUCTUATE [34]. We conducted Bottleneck analyses under the infinite allele model (IAM; [35]), stepwise mutation model (SMM; [36]), and two-phase model of mutation (TPM; [37]). Parameters for the TPM were set at 80% SMM with a variance of 9% [38,39], with 1,000 simulations performed for each population. Significance was assessed using a Wilcoxon sign-rank test and following application of Bonferroni adjustments to critical α values. Significant heterozygosity deficit values relative to the number of alleles indicate recent population growth, whereas heterozygosity excess relative to the number of alleles indicates a recent population bottleneck [33]. We note that BOTTLENECK compares heterozygosity deficiency and excess relative to number of alleles expected at mutation-drift equilibrium, and not to HWE expectation [33]. To allow assessment of recent population demography using sufficient numbers of representative individuals, we pooled data from TAN, POR, and YUK into a single population, and similarly NEAR and COMM.

FLUCTUATE estimates a population growth parameter, *g*, incorporating coalescence theory (parameters: 10 short chains, sampling increments of 10 with 1,000 steps per chain; 10 long chains, sampling increments of 10 with 20,000 steps per chain; a random starting tree; and a starting value of *g* set to 1). Data were analyzed 5 times, and parameters converged across runs. Positive values of *g* suggest population growth over time; negative values suggest population decline. As this method incorporates aspects of genealogy, it is sensitive to changes in demography and thus may be upwardly biased [34]. As a result, and because standard deviations are only approximate, we used *g* to indicate population growth if *g* > 3 SD(*g*). Fluctuations in historical population size were also inferred from Tajima’s *D* and Fu’s *F*_S_ (see above), generated using ARLEQUIN.

**References**

1. Sambrook J, Fritsch EF, Maniatis T. Molecular cloning: a laboratory manual. 2nd ed. Cold Spring Harbor, NY: Cold Spring Harbor Laboratory Press; 1989.

2. Medrano JF, Aasen E, Sharrow L. DNA extraction from nucleated red blood cells. Biotechniques. 1990;8: 43.

3. Sonsthagen SA, Talbot SL, White CM. Gene flow and genetic characterization of Northern Goshawks breeding in Utah. Condor. 2004;106: 826–836. doi:10.1650/7448

4. Talbot SL, Palmer AG, Sage GK, Sonsthagen SA, Swem T, Brimm DJ, et al. Lack of genetic polymorphism among peregrine falcons *Falco peregrinus* of Fiji. J Avian Biol. 2011;42: 415–428. doi:10.1111/j.1600-048X.2011.05280.x

5. Nesje M, Røed KH, Lifjeld JT, Linberg P, Steen O. Genetic relationships in the peregrine falcon (*Falco peregrinus*) analysed by microsatellite DNA markers. Mol Ecol. 2000;9: 53–60.

6. Brown JW, van Coeverden de Groot PJ, Birt TP, Seutin G, Boag PT, Friesen VL. Appraisal of the consequences of the DDT-induced bottleneck on the level and geographic distribution of neutral genetic variation in Canadian peregrine falcons, *Falco peregrinus*. Mol Ecol. 2007;16: 327–343. doi:10.1111/j.1365-294X.2007.03151.x

7. Johnson JA, Talbot SL, Sage GK, Burnham KK, Brown JW, Maechtle TL, et al. The use of genetics for the management of a recovering population: temporal assessment of migratory peregrine falcons in North America. PLoS One. 2010;5: e14042. doi:10.1371/journal.pone.0014042

8. White CM, Sonsthagen SA, Sage GK, Anderson C, Talbot SL. Genetic relationships among some subspecies of the Peregrine Falcon (*Falco peregrinus* L.), inferred from mitochondrial DNA control-region sequences. Auk. 2013;130: 78–87. doi:10.1525/auk.2012.11173

9. Bandelt H-J, Forster P, Sykes BC, Richards MB. Mitochondrial portraits of human populations using median networks. Genetics. 1995;141: 743–753.

10. Rousset F. GENEPOP’007: a complete re-implementation of the GENEPOP software for Windows and Linux. Mol Ecol Resour. 2008;8: 103–106. doi:10.1111/j.1471-8286.2007.01931.x

11. Petit RJ, el Mousadik A, Pons O. Identifying populations for conservation on the basis of genetic markers. Conserv Biol. 1998;12: 844–855.

12. Goudet J. FSTAT: a program to estimate and test gene diversities and fixation indices [Internet]. 2001. Available: http://www2.unil.ch/popgen/softwares/fstat.htm

13. Excoffier L, Laval G, Schneider S. Arlequin (version 3.0): an integrated software package for population genetics data analysis. Evol Bioinform Online. 2005;1: 47–50. doi:10.1111/j.1755-0998.2010.02847.x

14. Fu Y-X. Statistical tests of neutrality of mutations against population growth, hitchhiking and background selection. Genetics. 1997;147: 915–925.

15. Tajima F. Statistical method for testing the neutral mutation hypothesis by DNA polymorphism. Genetics. 1989;123: 585–595. doi:PMC1203831

16. Zeng K, Fu Y-X, Shi S, Wu C-I. Statistical tests for detecting positive selection by utilizing high-frequency variants. Genetics. 2006;174: 1431–1439. doi:10.1534/genetics.106.061432

17. Tajima F. The effect of change in population size on DNA polymorphism. Genetics. 1989;123: 597–601.

18. Weir BS, Cockerham CC. Estimating F-statistics for the analysis of population structure. Evolution (N Y). 1984;38: 1358–1370.

19. Slatkin M. A measure of population subdivision based on microsatellite allele frequencies. Genetics. 1995;139: 457–462.

20. Fisher RA. Statistical methods for research workers. 14th ed. Edinburgh: Oliver and Boyd; 1970.

21. Manly BFJ. The statistics of natural selection on animal populations. London: Chapman and Hall; 1985.

22. Pritchard JK, Stephens M, Donnelly P. Inference of population structure using multilocus genotype data. Genetics. 2000;155: 945–959. doi:10.1111/j.1471-8286.2007.01758.x

23. Evanno G, Regnaut S, Goudet J. Detecting the number of clusters of individuals using the software STRUCTURE: a simulation study. Mol Ecol. 2005;14: 2611–2620. doi:10.1111/j.1365-294X.2005.02553.x

24. Posada D, Crandall KA. MODELTEST: testing the model of DNA substitution. Bioinformatics. 1998;14: 817–818. doi:10.1063/1.2218048

25. Excoffier L, Smouse PE, Quattro JM. Analysis of molecular variance inferred from metric distances among DNA haplotypes: application to human mitochondrial DNA restriction data. Genetics. 1992;131: 479–491. doi:10.1007/s00424-009-0730-7

26. Goudet J, Raymond M, De Meeüs T, Rousset F. Testing differentiation in diploid populations. Genetics. 1996;144: 1933–1940. doi:10.1111/j.1471-8286.2007.01769.x

27. Cavalli-Sforza LL, Edwards AWF. Phylogenetic analysis. Models and estimation procedures. Am J Hum Genet. 1967;19: 233–257. Available: http://www.pubmedcentral.nih.gov/articlerender.fcgi?artid=1706274&tool=pmcentrez&rendertype=abstract

28. Saitou N, Nei M. The neighbor-joining method: a new method for reconstructing phylogenetic trees. Mol Biol Evol. 1987;4: 406–425.

29. Langella O. Populations 1.2.30: population genetic software [Internet]. Gif-sur-Yvette, France: Laboratoire Evolution, Génomes et Spéciation; 1999. Available: http://bioinformatics.org/~tryphon/populations/

30. Page RDM. TreeView: an application to display phylogenetic trees on personal computers. Comput Appl Biosci CABIOS. 1996;12: 357–358. doi:10.1093/bioinformatics/12.4.357

31. Beerli P, Felsenstein J. Maximum-likelihood estimation of migration rates and effective population numbers in two populations using a coalescent approach. Genetics. 1999;152: 763–773. doi:10.1073/pnas.081068098

32. Beerli P, Felsenstein J. Maximum likelihood estimation of a migration matrix and effective population sizes in n subpopulations by using a coalescent approach. Proc Natl Acad Sci U S A. 2001;98: 4563–4568. doi:10.1073/pnas.081068098

33. Cornuet JM, Luikart G. Description and power analysis of two tests for detecting recent population bottlenecks from allele frequency data. Genetics. 1996;144: 2001–2014.

34. Kuhner MK, Yamato J, Felsenstein J. Maximum likelihood estimation of population growth rates based on the coalescent. Genetics. 1998;149: 429–434.

35. Maruyama T, Fuerst PA. Population bottlenecks and nonequilibrium models in population genetics. II. Number of alleles in a small population that was formed by a recent bottleneck. Genetics. 1985;111: 675–689.

36. Ohta J, Kimura M. A model of mutation appropriate to estimate the number of electrophoretically detectable alleles in a finite population. Genet Res. 1973;22: 201–204.

37. Di Rienzo A, Peterson AC, Garza JC, Valdes AM, Slatkin M, Freimer NB. Mutational processes of simple-sequence repeat loci in human populations. Proc Natl Acad Sci U S A. 1994;91: 3166–3170. doi:10.1073/pnas.91.8.3166

38. Garza JC, Williamson EG. Detection of reduction in population size using data from microsatellite loci. Mol Ecol. 2001;10: 305–318.

39. Piry S, Luikart G, Cornuet J-M. BOTTLENECK: a computer program for detecting recent reductions in the effective population size using allele frequency data. J Hered. 1999;90: 502–503. doi:10.1093/jhered/90.4.502
