# Supplementary figures and images for: Intraspecific evolutionary relationships among peregrine falcons in western North American high latitudes
Source: PLoS One. 2017 Nov 17;12(11):e0188185. doi: 10.1371/journal.pone.0188185 (PMC5693296; doi:10.1371/journal.pone.0188185)

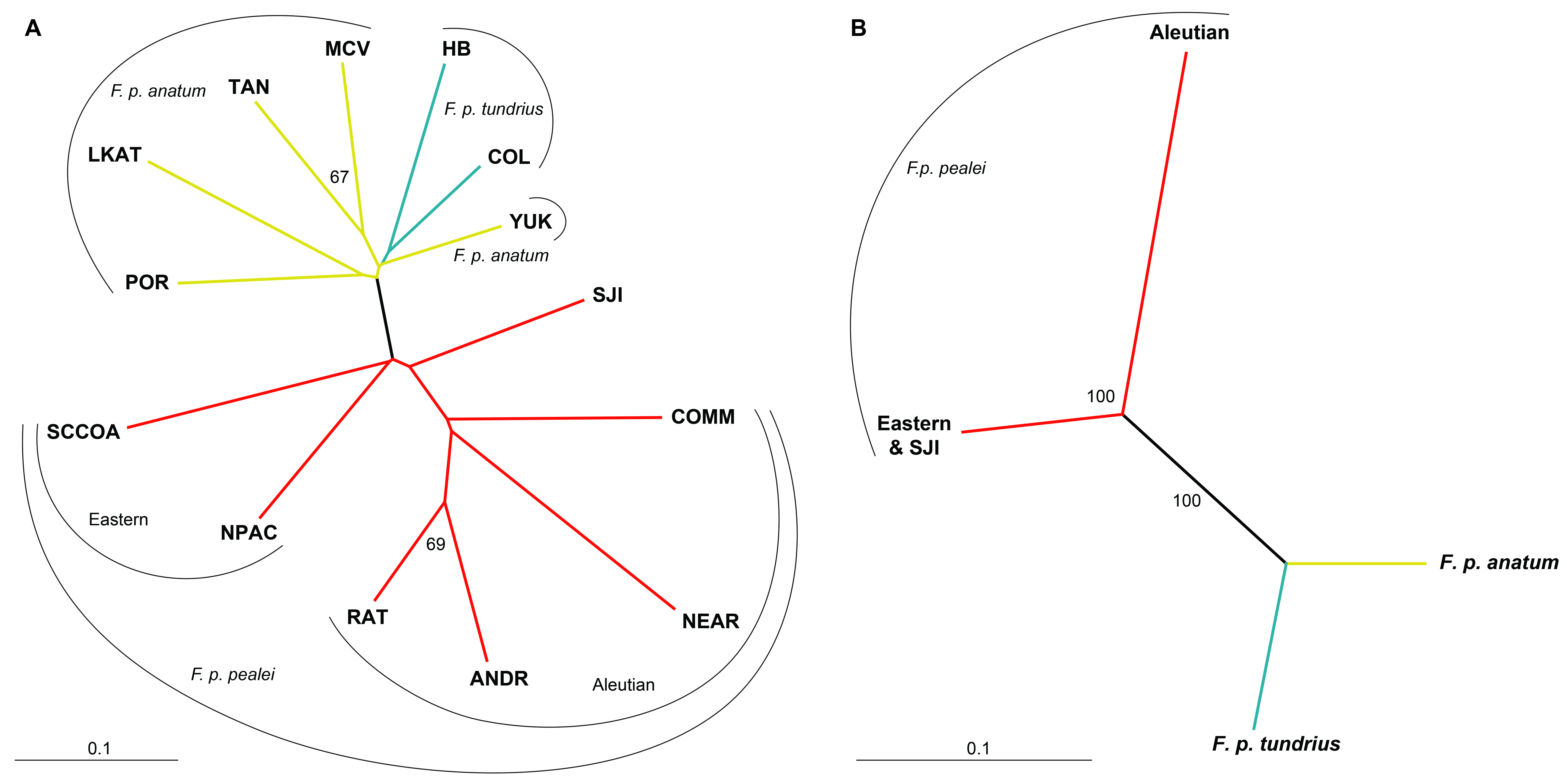

Supplement: S1 Fig — Illustrated are relationships among A) 14 populations of peregrine falcon in high latitudes habitats in Canada, Alaska and the Commander Islands, Russia; and B) three subspecies (F. p. anatum, F. p. tundrius, and F. p. pealei). Populations within the distribution of F. p. pealei are designated with red branches, within the distribution of F. p. anatum with yellow branches, and within the distribution of F. p. tundrius with blue branches. Values in nodes designate bootstrap values, based on 1000 permutations (only values > 50% are shown). (TIF) [file pone.0188185.s002.tif]
