# Supplementary material for: Intraspecific evolutionary relationships among peregrine falcons in western North American high latitudes
Source: PLoS One. 2017 Nov 17;12(11):e0188185. doi: 10.1371/journal.pone.0188185 (PMC5693296; doi:10.1371/journal.pone.0188185)
Supplement: S1 Table — Shown are values for pairwise χ2 (above diagonal) and FST (below diagonal) comparisons among populations of F. peregrinus within Alaska, based on data from 11 microsatellite loci. See manuscript for locales associated with acronyms. Values in bold, and values listed as ∞ (= infinity) indicate significant differences in the distribution of alleles/haplotypes or variance in allelic/haplotypic frequency, respectively, following Bonferroni corrections (α = 0.0045). The population on the San Juan Islands (SJI) is considered a contact zone between continental populations (F. p. anatum) and maritime populations (F. p. pealei). (DOCX) [file pone.0188185.s003.docx]

**S1 Table**

|  | TAN | YUK | POR | LKAT | MCV | HB | COL | SJI | NPAC | SCCOA | ANDR | RAT | NEAR | COMM |
| --- | --- | --- | --- | --- | --- | --- | --- | --- | --- | --- | --- | --- | --- | --- |
| TAN | — | 19.464 | 32.436 | **44.422** | 19.864 | 35.707 | 31.861 | **63.618** | **54.310** | **72.773** | **∞** | **∞** | **∞** | **∞** |
| YUK | 0.006 | — | 17.016 | 29.524 | 25.757 | 40.489 | 29.529 | ∞ | **53.951** | **76.432** | **∞** | **∞** | **∞** | **∞** |
| POR | 0.058 | 0.013 | — | 37.535 | 33.454 | **48.753** | 29.032 | ∞ | **58.517** | **75.734** | **∞** | **∞** | **73.746** | **∞** |
| LKAT | 0.076 | 0.035 | 0.053 | — | **46.990** | **59.683** | 38.591 | **78.931** | **58.602** | **70.672** | **∞** | **∞** | **∞** | **61.605** |
| MCV | 0.006 | 0.013 | 0.052 | 0.071 | — | 28.973 | 35.745 | **69.280** | **66.404** | **60.162** | **∞** | **∞** | **∞** | **66.798** |
| HB | 0.034 | 0.018 | **0.065** | **0.106** | 0.025 | — | **63.553** | **∞** | **71.813** | **97.063** | **∞** | **∞** | **∞** | **81.476** |
| COL | 0.026 | 0.005 | 0.031 | 0.042 | 0.029 | **0.033** | — | **∞** | **89.222** | **∞** | **∞** | **∞** | **∞** | **∞** |
| SJI | **0.085** | **0.086** | **0.123** | **0.103** | **0.080** | **0.115** | **0.106** | — | **67.311** | **76.790** | **∞** | **∞** | **46.930** | **∞** |
| NPAC | **0.088** | **0.054** | **0.097** | **0.091** | **0.103** | **0.070** | **0.071** | **0.067** | — | **59.809** | **∞** | **∞** | **47.711** | **51.622** |
| SCCOA | **0.104** | **0.067** | **0.100** | **0.114** | **0.075** | **0.092** | **0.101** | **0.062** | **0.077** | — | **83.281** | **99.942** | **43.322** | **62.220** |
| ANDR | **0.217** | **0.181** | **0.214** | **0.219** | **0.175** | **0.200** | **0.198** | **0.120** | **0.127** | **0.095** | — | 30.244 | 34.574 | **∞** |
| RAT | **0.200** | **0.160** | **0.193** | **0.168** | **0.155** | **0.172** | **0.174** | **0.108** | **0.092** | **0.107** | **0.011** | — | 31.451 | **∞** |
| NEAR | **0.225** | **0.174** | **0.245** | **0.175** | **0.184** | **0.244** | **0.180** | 0.089 | **0.136** | **0.111** | **0.040** | **0.029** | — | 38.041 |
| COMM | **0.141** | **0.099** | **0.117** | **0.129** | **0.113** | **0.127** | **0.121** | **0.102** | **0.091** | **0.089** | **0.076** | **0.093** | **0.088** | — |
