# Supplementary material for: Intraspecific evolutionary relationships among peregrine falcons in western North American high latitudes
Source: PLoS One. 2017 Nov 17;12(11):e0188185. doi: 10.1371/journal.pone.0188185 (PMC5693296; doi:10.1371/journal.pone.0188185)
Supplement: S2 Table — Populations augmented with non-native subspecies following the population decline are shaded. (DOCX) [file pone.0188185.s004.docx]

**S2 Table**

|  | ***F. p. pealei*** | | | | | | |  | ***unknown*** |  | ***F. p. anatum*** | | | | | |  | ***F. p. tundrius*** | | |
| --- | --- | --- | --- | --- | --- | --- | --- | --- | --- | --- | --- | --- | --- | --- | --- | --- | --- | --- | --- | --- |
| **Haplotype** | **Eastern** | |  | **Aleutian Islands** | | | |  |  |  |  | | |  |  | |  |  | |  |
|  | **NPAC** | **SCCOA** |  | **ANDR** | **RAT** | **NEAR** | **COMM** |  | **SJI** |  | **TAN** | **YUK** | **POR** |  | **LKAT** | **MCV** |  | **COL** |  | **HB** |
| **CR2** | 7 | 6 |  | 10 | 12 | 4 | 4 |  | 7 |  | 7 | 12 | 7 |  | 2 | 7 |  | 27 |  | 10 |
| **CR3** | - | - |  | - | 1 | - | - |  | - |  | - | - | - |  | - | - |  | - |  | - |
| **CR4** | - | - |  | - | - | - | - |  | - |  | 1 | 2 | - |  | 1 | - |  | 7 |  | 2 |
| **CR5** | - | 1 |  | - | - | - | - |  | - |  | - | 1 | - |  | - | 1 |  | - |  | - |
| **CR6** | 2 | - |  | - | 1 | - | - |  | 4 |  | - | - | - |  | - | - |  | 3 |  | - |
| **CR9** | - | - |  | - | - | - | 1 |  | 2 |  | - | - | - |  | - | - |  | - |  | - |
| **CR10** | - | 5 |  | - | - | - | - |  | 1 |  | - | - | - |  | - | - |  | - |  | - |
| **CR11** | - | - |  | - | - | - | - |  | - |  | - | - | - |  | - | - |  | 1 |  | - |
| **CR12** | - | - |  | - | - | - | - |  | - |  | - | - | - |  | - | - |  | 1 |  | - |
| **CR14** | - | - |  | - | - | - | - |  | - |  | 1 | - | - |  | - | - |  | - |  | 1 |
| **CR17** | - | - |  | - | - | - | - |  | 1 |  | - | - | - |  | - | - |  | - |  | - |
| **CR29** | - | - |  | - | - | - | - |  | - |  | - | - | - |  | - | - |  | 1 |  | - |
| **CR37** | - | - |  | - | - | - | - |  | - |  | - | - | - |  | - | - |  | 1 |  | - |
| **CR40** | - | - |  | - | 1 | - | - |  | - |  | - | - | - |  | - | - |  | - |  | - |
| **CR48** | - | - |  | - | - | - | - |  | - |  | - | - | - |  | 3 | - |  | - |  | - |
| **Total** | 9 | 12 |  | 10 | 15 | 4 | 5 |  | 15 |  | 9 | 15 | 7 |  | 6 | 8 |  | 41 |  | 13 |
