# Supplementary material for: Intraspecific evolutionary relationships among peregrine falcons in western North American high latitudes
Source: PLoS One. 2017 Nov 17;12(11):e0188185. doi: 10.1371/journal.pone.0188185 (PMC5693296; doi:10.1371/journal.pone.0188185)
Supplement: S3 Table — Shown are values for pairwise χ2 (above diagonal) and ϕST (below diagonal) comparisons among populations of F. peregrinus within Alaska, based on sequence data from the mitochondrial DNA control region. See manuscript for locales associated with acronyms. Values in bold or (for χ2) designated as ∞ (infinity) indicate significant differences in the distribution of haplotypes or variance in haplotype frequency (P < 0.05), respectively. The population on the San Juan Islands (SJI) is considered a contact zone between continental populations (F. p. anatum) and coastal populations (F. p. pealei). (DOCX) [file pone.0188185.s005.docx]

**S3 Table**

|  | TAN | YUK | POR | LKAT | MCV | HB | COL | SJI | NPAC | SCCOA | ANDR | RAT | NEAR | COMM |
| --- | --- | --- | --- | --- | --- | --- | --- | --- | --- | --- | --- | --- | --- | --- |
| TAN | — | 3.072 | 2.837 | **12.901** | 4.320 | 0.000 | 5.048 | **15.085** | **7.398** | **17.245** | **6.377** | **7.244** | 1.991 | 4.546 |
| YUK | -0.047 | — | 3.827 | **15.915** | 2.196 | 2.477 | **6.193** | **∞** | **10.618** | **19.807** | 5.090 | **10.154** | 2.531 | **6.893** |
| POR | -0.034 | -0.024 | — | **14.940** | 1.452 | 3.854 | 4.470 | 3.791 | 4.427 | **12.294** | n/a | 2.043 | n/a | 3.651 |
| LKAT | **0.219** | **0.268** | 0.325 | — | **14.975** | **15.369** | **∞** | **∞** | **21.640** | **18.230** | **23.026** | **10.829** | **10.402** | **12.351** |
| MCV | -0.002 | -0.033 | -0.018 | 0.285 | — | **6.562** | **9.179** | **14.837** | 5.931 | **12.574** | 3.329 | 4.623 | 1.250 | 4.004 |
| HB | -0.095 | -0.047 | -0.011 | **0.241** | 0.024 | — | 5.246 | **∞** | 10.321 | **∞** | 5.119 | **9.743** | 2.685 | **6.830** |
| COL | 0.004 | 0.011 | 0.024 | **0.211** | 0.054 | 0.002 | — | **∞** | 5.417 | **∞** | **7.815** | **12.674** | 2.015 | **7.577** |
| SJI | **0.193** | **0.232** | 0.175 | **0.158** | **0.174** | **0.227** | **0.175** | — | 5.030 | **∞** | **17.748** | **14.237** | **6.206** | 4.611 |
| NPAC | 0.067 | 0.093 | 0.087 | **0.291** | **0.080** | 0.091 | 0.014 | 0.041 | — | **17.896** | **6.406** | 3.013 | 2.575 | **6.233** |
| SCCOA | **0.326** | **0.366** | 0.309 | **0.190** | **0.272** | **0.371** | **0.404** | 0.103 | **0.265** | — | **17.748** | **∞** | **7.207** | **11.259** |
| ANDR | 0.009 | 0.010 | 0.000 | **0.404** | **0.029** | 0.027 | 0.051 | **0.220** | 0.141 | 0.362 | — | 3.637 | n/a | 4.524 |
| RAT | 0.007 | 0.019 | -0.044 | 0.144 | **-0.042** | 0.036 | 0.047 | 0.007 | -0.055 | 0.258 | -0.011 | — | 2.200 | 5.553 |
| NEAR | -0.119 | -0.098 | 0.000 | 0.203 | -0.109 | -0.088 | 0.037 | 0.100 | -0.006 | 0.228 | 0.000 | -0.117 | — | 1.481 |
| COMM | 0.038 | 0.085 | 0.073 | -0.011 | **0.054** | 0.074 | 0.016 | -0.056 | -0.087 | 0.131 | 0.149 | -0.100 | -0.053 | — |
